# The prevalence of food insecurity and its relationship with wellbeing in a large, cross‐sectional study of children and young people in England

**DOI:** 10.1002/jcv2.70049

**Published:** 2025-09-23

**Authors:** Giacomo Bignardi, Mina Fazel, OxWell Study Team, Sarah‐Jayne Blakemore

**Affiliations:** ^1^ Social, Genetic & Developmental Psychiatry Centre Institute of Psychiatry, Psychology & Neuroscience King's College London London UK; ^2^ Department of Psychology University of Cambridge Cambridge UK; ^3^ Department of Psychiatry University of Oxford Oxford UK

**Keywords:** adolescence, depression, food insecurity, mental health, wellbeing

## Abstract

**Background:**

We aimed to assess the prevalence of food insecurity reported by children and young people in four areas of England in 2023 and examine its association with mental health and wellbeing.

**Methods:**

We used data from the OxWell student survey, a large, diverse, cross‐sectional study of 38,430 students aged 8–19 years, conducted primarily in four counties in England (Berkshire, Buckinghamshire, Merseyside, Oxfordshire) during February and March 2023. Students responded to three food insecurity questions and completed a battery of mental health and wellbeing questionnaires. Analysis plans were pre‐registered prior to data access. Bayesian mixed‐effect ordinal regression models were used to estimate associations with outcomes, controlling for gender, school year, ethnicity, birth location, parental birth location, deprivation and school.

**Results:**

Depending on the question, 4%–6% of children and young people reported *sometimes* experiencing food insecurity, and 1%–2% reported often experiencing it. After controlling for covariates, high levels of food insecurity were associated with increased depression (SMD = 0.51) and anxiety scores (SMD = 0.36), reduced adolescent wellbeing (SMD = −0.44) and positive thoughts (SMD = −0.30). Food insecurity's association with loneliness (SMD = 0.20, 99% CI [−0.03, 0.42]) and child wellbeing (SMD = 0.40, 99% CI [−0.00, 0.79]) were less certain in direction.

**Conclusions:**

Food insecurity remains a persistent problem facing children and young people in England and is associated with deteriorated wellbeing. Our findings underscore the urgent need to identify and support families struggling with the rising cost of living.

## Key points

**What's known?**

- Food insecurity affects physical health and development, but there's limited recent research on young people's direct experiences and mental health impacts in England.

**What's new?**

- Large cross‐sectional survey of 38,430 children and adolescents aged 8–19 in England.
- 5%–8% of participants reported experiencing food insecurity sometimes or often.
- Food insecurity, particularly going to bed hungry, was associated with worsened mental health and wellbeing, with effects ranging from small to medium.

**What's relevant?**

- Findings emphasize the importance of identifying and supporting children and young people at risk of food insecurity during the ongoing cost‐of‐living crisis.

## INTRODUCTION

Like many other nations, the United Kingdom (UK) has recently experienced rapid increases in energy, housing and food costs, fuelling a cost‐of‐living crisis (Joseph Rowntree Foundation, 2024). Concerns have intensified regarding the number of children and young people growing up in households that struggle to afford basic necessities, and the potential impact of material deprivation and food insecurity on children and young people's mental health and wellbeing. Food insecurity is usually defined on a continuum, from moderate levels, involving a reduction in food quality, variety or desirability to more severe levels, involving disrupted or insufficient nutrition (Knowles et al., 2016). The widely‐used USDA Adult Food Security module measures food insecurity through self‐reported reduction of quality and size of meals, hunger, weight loss and perceived food affordability. Food insecurity is tightly associated with household income levels (Food Standards Agency, 2023, p. 5; UK Government, 2023), and often co‐occurs with housing and energy insecurity and other adverse childhood experiences (Dermott & Pomati, 2017; Padgett & Fay, 2023). This can make it difficult to isolate the specific effect of food insecurity from the myriad consequences of poverty (Bignardi et al., 2022). The importance of nutrition for physical development and health is well‐documented (Thomas et al., 2019). However, there remains a notable gap in research exploring this young generation's experiences of food insecurity and its impact on their wellbeing. Using data from a large survey of students in England (OxWell 2023 Student Survey), we aimed to quantify the prevalence of experienced food insecurity among children aged 8–19 years and assess its association with mental health and wellbeing.

Although household food insecurity is typically measured through parent report, children and young people's experiences provide a valuable additional perspective. Many parents shield their children and young people from hardship, for example, by cutting back on their own food and necessities. Thus, children and young people may be less likely to experience food insecurity than parents (Nord, 2013). Children and young people's experiences of food insecurity differ from those of adults, but they may be more aware of household food insecurity (Fram et al., 2011; Knowles et al., 2016; Leung et al., 2020) than their parents think (Fram et al., 2011; Frongillo et al., 2019). While adults' experience of food insecurity is often centred around affordability, children and young people's experiences are typically centred around the immediate social and food environment, such as worry about the availability of preferred food and wellbeing, stress in household relationships and embarrassment in front of friends (Fram et al., 2011; Leung et al., 2020).

Until recently, few studies had explored the prevalence of food insecurity in the UK. Several national surveys have started collecting adult‐reported household food insecurity data. Data from the 2021 to 2022 Family Resources Survey indicates that 6% of households in the UK experienced low or very low food security, and 6% had marginal food security, according to the USDA scale (UK Government, 2023). Data from the 2022 fifth wave of the Food and You national survey also indicated that 9% of households in England were classified as very food insecure, and this rate was higher among adolescents (16 to 24‐year‐olds) and in households with children (Armstrong et al., 2023). The 2023 Winter Survey found that 4% of adults in Great Britain reported running out of food due to affordability in the past 2 weeks (Leach et al., 2024).

Existing research linking household food insecurity with mental health and wellbeing has revealed mixed findings. One study of 6483 North American 13 to 17‐year‐olds found a small but significant association between adult‐reported food insecurity and the probability of an adolescent having a mood and anxiety disorder, even when controlling for socioeconomic status (McLaughlin et al., 2012). Similarly, a study of 2123 English twins aged 7–10 found that after controlling for household income, parent‐reported food insecurity was associated with emotional problems in children, though not behavioural problems or IQ (Belsky et al., 2010). One meta‐analysis identified 19 studies testing cross‐sectional associations between food insecurity and mental health in adults, indicating a moderate average increase in depression and stress, but not anxiety, though the results from the included studies were highly inconsistent (Pourmotabbed et al., 2020). We identified only one previous large‐scale study assessing the association between adolescent‐reported food insecurity and mental health problems in Kenya. They found that adolescent (13 to 17‐year‐olds)—but not adult‐reported food insecurity was associated with a two‐item measure of general mental health.

Food insecurity may impact peer relationships, an important determinant of adolescent wellbeing (Tomova et al., 2021). Evidence suggests that childhood poverty can lead to social exclusion and the inability to participate in typical relationships and activities, such as lunchtimes, sports, drama and after school clubs, and other extra‐curricular and enriching activities (Crous & Bradshaw, 2017; Main & Bradshaw, 2017). One qualitative study found that some children in food insecure households worry about inviting friends to their house and being teased by others (Leung et al., 2020). Food insecure children and adolescents are more likely to perpetrate, and be victims of, bullying (Edwards & Taub, 2017; Fram et al., 2022). These factors may lead to problems with social relationships and increased loneliness, which are linked with a wide range of adverse outcomes in adolescence (Orben et al., 2020). There is, however, a paucity of research on the relationship between food insecurity and loneliness in adolescence. One previous study using Global Student Health Survey data found a small relationship between food insecurity and loneliness (Wu et al., 2022). Given the importance of peer relationships for adolescent development and the potential for food insecurity to strain these connections, we included self‐reported loneliness as an additional outcome variable in our study.

Here, we utilised the fourth wave of the OxWell Student Survey, a large‐scale, representative survey of 8 to 19‐year‐old primary, secondary and further education college (FEC) students in England in 2023. This was the first wave to include questions on food insecurity. Our first research aim was to assess the prevalence of child and young person‐reported food insecurity in our sample. Our second aim was to test whether higher levels of food insecurity are associated with reduced mental health and wellbeing after controlling for demographic and deprivation covariates. We controlled for potential confounds such as gender and ethnicity, which are well‐documented risk factors for food insecurity and mental health outcomes (Daly, 2022; Hallward et al., 2023; Yau et al., 2020). We hypothesised that increased levels of food insecurity would be associated with heightened symptoms of depression, anxiety and loneliness and with diminished wellbeing and positive thoughts.

## METHODS

### Study design

The 2023 OxWell Student Survey is a repeated cross‐sectional survey examining the health and wellbeing of students in school years 5–13 (approximately aged 8–19 years) in primary and secondary schools or FECs in England. The survey was approved by the University of Oxford Medical Sciences Interdivisional Research Ethics Committee (Reference: R62366/RE014) on 19 January 2023. Data collection took place between February and March 2023.

### Participants

Between February and March 2023, mainstream state‐maintained and independent schools in nine local educational authority catchment areas were invited to participate. These schools were primarily located in 4 English counties: Berkshire, Buckinghamshire, Merseyside and Oxfordshire. In total, 105 primary schools, 70 secondary schools and 10 FECs participated. Schools received a summary report and access to an OxWell data portal in return for participating.

Students were enroled using a parental opt‐out model along with students' assent (student age <16 years) or students' informed consent only (student age ≥16 years). The survey was self‐report and administered during the school day as part of in‐school activities and overseen by school staff. Students were shown a video and presentation explaining the survey, and teachers were present throughout administration and available to respond to any questions. OxWell was designed to maximise participation and representativeness and encourage more accurate responses by using a parental opt‐out consent model and not collecting identifiable student data.

### Procedures

All measures are outlined in Table 1. Some outcome variables were administered only to primary school students and others to secondary school/FEC students. Longer versions of revised children's anxiety and depression scale (RCADS) and the current 11‐item version have been validated for use in children as young as 8 years of age, demonstrating good reliability and validity (Carlander et al., 2024; Grothus et al., 2025; Klaufus et al., 2020). All explanatory variables were the same across groups. Food insecurity was measured using three questions about family food bank usage (F1), the means to afford to eat at school (F2), and going to bed hungry because of a lack of food in the household (F3; see Table 1). Participants could respond ‘never or hardly ever’, ‘some of the time’ or ‘often’ to each question. Polychoric correlations between these items were high (>0.5; see Supplement S1). All explanatory variables were dummy coded, including the food insecurity questions, deprivation questions and other covariates. For example, the food insecurity question ‘My family uses food banks’ (F1) was dummy coded into two variables (F1_sometimes, F1_often). ‘Prefer not to say’ responses were coded as missing.

**TABLE 1:** Explanatory and outcome variables.

Explanatory variables			
Variable code	Variable name	Dummy coded categories	Dummy coding reference group
Food insecurity questions			
F1	My family uses food banks	Some of the time	‘Never or hardly ever’ reference
F2	At school, I am unable to afford to eat		
F3	At home, I go to bed hungry because there is not enough food in the house	Often	
Covariates			
Gender	What is your gender?	Gender diverse (see main text)	‘Female’ reference
		Male	*‘Prefer Not to Say’ coded as missing*
Ethnicity	What is your ethnic group?	Asian	‘White’ reference
		Black	
		Mixed	
		Other	
Birth location	Were you born in the UK?	No	‘Yes’ reference
			*‘Prefer not to say’ coded as missing*
Parent birth location	Were your parents born in the UK?	No	‘Yes, one parent’ and ‘Yes, both parents’ combined into single reference group
			*‘Prefer not to say’ coded as missing*
School ID	Each educational institution was assigned a unique number	N/A	N/A
School year	School year group (integers between 5 and 13)	Youngest year group was used as dummy coding reference	N/A
Deprivation questions			
X1430	I worry about not having enough money for the things my family needs, for example, food, bills, electric or gas	Some of the time Often	‘Never or hardly ever’ reference
X1450	The house I live in is cold and/or damp		
X1460	At school, I am unable to afford the right uniform, games kit, books, equipment, or go on trips		
X1480	At home, I do not have enough space to do things like homework or chill out		
X1490	At home, I have no internet access or poor internet access		

Outcome variables			
	Information	Sampling frame	Reliability (%)
RCADS‐11 depression	RCADS‐11 Depression Subscale (Radez et al., 2021)	All	87
RCADS‐11 anxiety	RCADS‐11 Anxiety Subscale (Radez et al., 2021)	All	88
SWEMWBS adolescent wellbeing	Short Warwick‐Edinburgh Mental Wellbeing Scale (McKay & Andretta, 2017)	Secondary and FECs	87
Positive thoughts	Items: I am worthwhile, I can succeed, I rise to the challenge, I can do things as well as anyone else, I can have fun, I can relax, I am a good person, I am helpful	Secondary and FECs	94
Loneliness	UCLA Loneliness Scale (Adapted Short Form; Geulayov, 2022)	Secondary and FECs	88
SCWBS child wellbeing	Stirling Children’s Wellbeing Scale (SCWBS; Liddle & Carter, 2015)	Primary	91

*Note*: Reliability was calculated using coefficient alpha, using pairwise correlation matrices to account for missing data. Some outcome variables were administered to primary school pupils only or only secondary school and FECs students.

Abbreviation: RCADS, revised children's anxiety and depression scale.

If students responded ‘other’ to the gender question (see Table 1), they could enter a free text response under ‘Prefer to self‐identify’. The free‐text responses were screened to filter out likely disingenuous responses (Soneson et al., 2024); these gender responses were coded as missing. Other responses were categorized as gender diverse and included as a dummy variable in analyses.

All outcome variables were calculated by sum‐scoring the questionnaire items, following any necessary reverse scoring.

### Missing data and imputation

Descriptive information on missing data is available in the Supplement S2. For research question 2, we generated 100 imputed datasets using multivariate imputation by chained equations (MICE) and the predictive mean matching algorithm from the MICE R package (Buuren & Groothuis‐Oudshoorn, 2011). Imputation was performed separately within each school year. All explanatory variables and outcomes were included in the imputation model, including individual questionnaire items, but excluding school year and school ID. Following imputation, outcome variables were sum‐scored from the relevant imputed questionnaire items, and explanatory variables were dummy coded.

### Statistical analysis

#### Pre‐registration of statistical analysis

The analysis plan was pre‐registered before data access was given to S.‐J.B. and G.B. (Bignardi et al., 2023). All deviations from the pre‐registration are described in the Supplement S3. For research question 1, we modified the variables used to calculate weights due to data availability constraints. Because of concerns about whether younger children fully understood the food insecurity questions, weighted prevalence estimates are presented both with and without data from children in primary schools. For research question 2, posterior predictive checks indicated that regression models with normally distributed residuals poorly captured the distribution of outcome variables, which showed notable floor and ceiling effects. To address this issue, we used ordinal regression models instead.

##### Research question 1

First, we estimated the proportion of *never*, *sometimes* and *often* responses to each food insecurity question in the complete sample. Proportions and 99% credible intervals were calculated using Bayesian multinomial logistic regression.

Second, we estimated the weighted proportion of students reporting each dimension of food insecurity, weighted by school year and school funding (state or independent), as detailed in the Supplement S4. Post‐stratification weights were calculated using census data from 2023/2024 Department for Education (DfE) and the 2024 Independent Schools Council student census. We used census information on children and young people in England in school years 5–11 in state‐funded primary and secondary schools and independent (i.e., private) schools. Note that the DfE census only covers England and may miss some pupils, including those home‐schooled or in FECs. It is important to note that because we only sampled four areas in England, any differences in food insecurity rates in those areas may lead to biased prevalence estimates. Although weighting can partly correct selection bias, the absence of detailed socioeconomic data on the students and broader population limits the effectiveness of his approach. Weighted proportions were calculated using the Survey R package, along with 99% confidence intervals.

In the Supporting Information S1, we estimate which demographic factors were associated with food insecurity using ordinal logistic regression models (S5) and report descriptive statistics on food insecurity responses by school year group (S6).

##### Research question 2

Bayesian cumulative logistic ordinal regression models were used to estimate the relationship between food insecurity and outcomes while controlling for covariates, which are outlined in Table 1. Ordinal regression models were chosen because they better approximate the shape of sum‐score outcome variables, which are discrete, skewed and have ceiling and floor effects (Liddell & Kruschke, 2018). Models were fitted using the Hamiltonian Monte Carlo algorithm from the *Stan* programing language (see Supplement S7 for details). All categorical predictor variables were dummy coded, as outlined in Table 1. Each deprivation question was dummy coded and included as individual predictors in the model. No interactions between predictor variables were specified. Education setting (school ID) was entered into the model as a random intercept. We used weakly informative priors (described in the Supplement S7) such that inferences are driven primarily by the model and data—this approach is referred to as objective or calibrated Bayesian analysis (Berger et al., 2024; Little, 2006). We chose a Bayesian approach because the algorithm used by Stan is highly effective for fitting more complex models and includes extensive diagnostic tools to assess model convergence (Betancourt, 2018; Gelman, 2014). Additionally, Bayesian 99% credible intervals have a more intuitive probabilistic interpretation: there is a 99% probability that the population effect lies within the interval. This interpretation is not strictly valid for confidence intervals (McElreath, 2020; Nalborczyk et al., 2019).

We opted against deriving a food insecurity sum score as a predictor for several reasons. First, unlike using a sum score, dummy coding does not assume that each food insecurity question is equally important, and it allows for non‐linear effects of each response category (never, sometimes or often). This enables us to identify which aspects of food insecurity are most strongly associated with each outcome. For similar reasons, we also entered each deprivation question into the model individually using dummy coding, rather than via creating a single deprivation score. Second, regression coefficients from a sum score predictor can be difficult to interpret, as the scale is arbitrary. Standardised regression coefficients or variance explained metrics (*R* ^2^) cannot solve this issue and can even be misleading, because these metrics are sensitive to variance in the predictor (Baguley, 2009). Because most students in England do not experience food insecurity, its contribution to the variance explained (*R* ^2^) could be small, even if the absolute effects are very large. Instead, we use dummy coding and marginal effects (explained below) to estimate how mental health outcomes change at different, specific levels of food insecurity, tied to specific responses on the questions, rather than arbitrary scales.

In the Supplement S9, we run exploratory analyses evaluating if the effects of food insecurity differ by gender, ethnicity and school year, using multilevel models.

###### Marginal effects (Δ, SMD) and levels of food insecurity

In each model, food insecurity is represented by six dummy‐coded ordinal regression coefficients. For greater interpretability, we report marginal effects in the main text; the corresponding regression coefficients can be found in the Supplement S8. Marginal effects involve using our fitted statistical model to predict outcomes under different counterfactual scenarios—in our case, different food insecurity levels (Arel‐Bundock et al., 2024; Heiss, 2021). This allows us to present simple, interpretable statistics from complex statistical models.

We calculated marginal effects at three levels of food insecurity: low, medium and high. Each level of food insecurity is defined by a specific set of values on the explanatory variables, called a *reference grid.* An example reference grid is provided in the Supplement S7. For all levels of food insecurity, covariate values are fixed at their sample means. Low food insecurity was defined as responding *never* across all food insecurity questions, medium food insecurity was defined as responding *sometimes* on each question, and high food insecurity was defined as responding *often* to each question. These three food insecurity levels represent three counterfactual participants who are average on the covariates but have different food insecurity responses.

We calculated three different marginal effects: *predictions* of the outcome for each food insecurity level, *comparisons* (Δ) of predictions between different food insecurity levels, and standardised mean differences (SMD) comparing different food insecurity levels.

Predictions represent the outcome's expected value (i.e., its mean) at low, medium and high food insecurity levels. Predictions are estimated using the fitted ordinal model and reference grid. Comparisons (Δ) are the absolute differences in predictions between different food insecurity levels. We calculated comparisons (Δ) between low and medium food insecurity, and between low and high food insecurity. Because these statistics are based on the units of the outcome variable, we also reported the SMD, which can be interpreted similarly to Cohen's *d*. An SMD of ¼ indicates that the two food insecurity levels differ by ¼ of a standard deviation on the outcome. More detail on calculation is provided in the Supplement S7.

Posterior means and 99% highest density continuous credible intervals were calculated for all effects. To make statistical inferences about the presence or absence of an effect, we calculated the probability of direction (pd), which describes our certainty in the direction of the estimated effect (Makowski et al., 2019). A pd of 97.5% indicates that we can be 97.5% certain that the population effect is in the same positive or negative direction as the estimated effect. Estimates of pd > 97.5%, pd > 99.5% and pd > 99.95% correspond approximately to *p*‐values of *p* < 0.05, *p* < 0.01 and *p* < 0.001, respectively. We pre‐registered a threshold of pd > 99.5% to declare the presence of an effect (Bignardi et al., 2023).

It is important to reiterate that individuals in the sample are not categorised into each level of food insecurity; marginal effects are calculated *after* fitting the model. This approach maximises statistical precision relative to categorising the food insecurity variables before model fitting (e.g., Taylor & Yu, 2002). Because marginal effects are calculated from the regression model, the estimates are adjusted by the covariates controlled in the model.

#### Role of the funding source

The funding source had no involvement in the study.

## RESULTS

### Descriptive statistics

A total of 43,735 participants initiated the survey. Of these, 1520 did not complete consent questions and were excluded. A further 3785 were excluded either because they did not respond to any outcome variable or did not meet our minimal engagement thresholds (at least 10 min).

The final sample size was 38,430, demographically described in Table 2. The age and ethnicity distribution of the sample is provided in the Supplement S2. The overall distribution of student ethnicity was similar to available 2023 census data for state‐funded secondary schools (see Supplement S2). Table 3 presents descriptive statistics for each outcome variable, which are available stratified by food insecurity response in the Supplement S10.

**TABLE 2:** Demographic information.

Subgroup	Primary school		Secondary school^b^	
	*N*	%	*N*	%
Gender				
Female^a^	4203	50.58	14,712	51.64
Male	4072	49.01	13,395	47.02
Gender diverse	34	0.41	383	1.34
Missing data	387		1244	
Ethnicity				
White^a^	4836	67.27	15,753	65.20
Asian	1209	16.82	4139	17.13
Black	304	4.23	1417	5.86
Mixed	412	5.73	1654	6.85
Other	428	5.95	1199	4.96
Missing data	1507		5572	
Child born UK				
UK^a^	7309	86.55	24,499	84.48
Not UK	1136	13.45	4502	15.52
Missing data	251		733	
Parent born UK				
Yes, one parent^a^	1363	16.90	4175	14.63
Yes, both parents^a^	4477	55.50	16,047	56.22
Neither parent	2226	27.60	8320	29.15
Missing data	630		1192	
Total	8696	100.00	29,734	100.00

^a^ Reference group for dummy coding. Prefer not to say responses are coded as missing.

^b^ All students in school years 7 or above, including further education colleges and independent school students.

**TABLE 3:** Outcome variable descriptive statistics on imputed and non‐imputed data.

Outcome	Imputed data				Non‐imputed data		
	*N*	Complete (%)	*M*	SD	*N*	*M*	SD
Primary school students							
RCADS‐11 depression	8696	88.9	3.79	3.24	7752	3.79	3.28
RCADS‐11 anxiety	8696	88.9	5.02	4.15	7746	5.02	4.18
Child wellbeing SCWBS	8696	90.3	52.91	11.13	7852	52.92	11.26
Secondary school students							
RCADS‐11 depression	29,734	82.3	5.02	3.91	24,469	5.09	4.03
RCADS‐11 anxiety	29,734	82.3	5.55	4.67	24,478	5.64	4.79
Adolescent wellbeing SWEMWBS	29,734	83.7	21.95	5.87	24,847	21.95	6.01
Positive thoughts	29,734	65.7	19.39	8.05	19,486	19.38	8.50
Loneliness	29,734	90.4	2.88	2.47	26,574	2.89	2.49

*Note*: Complete column indicates the percentage of non‐missing values on individual questionnaire items. Non‐imputed summary scores were calculated for participants completing all questionnaire items.

Abbreviation: RCADS, revised children's anxiety and depression scale.

### Research question 1: Prevalence

Table 4 shows the prevalence of food insecurity across the whole sample, and post‐stratification weighted prevalence estimates across specific school year ranges. Descriptive statistics on food insecurity stratified by school year can be found in the Supplement S6. Across all students in the sample, around 92%–95% reported never experiencing food insecurity, depending on the question. Around four to 6% of students reported *sometimes* experiencing food insecurity, and around 1%–2% of students reported *often* experiencing food insecurity. The proportion of students *never* experiencing food insecurity was highest for question F3 (go to bed hungry). In the Supplement S5, we estimate which demographic factors were associated with food insecurity using ordinal logistic regression models, finding an association with gender, school year and ethnicity.

**TABLE 4:** Prevalence of food insecurity across the whole sample, and post‐stratification weighted prevalence.

Food insecurity question^a^	Never or hardly ever			Some of the time			Often		
	%	99% CI LB	99% CI UB	%	99% CI LB	99% CI UB	%	99% CI LB	99% CI UB
All students in sample (unweighted proportions)									
F1	92.7	92.3	93.0	5.8	5.4	6.1	1.6	1.4	1.7
F2	92.2	91.9	92.6	5.4	5.1	5.7	2.4	2.2	2.6
F3	94.5	94.2	94.8	4.2	3.9	4.4	1.4	1.2	1.5
Post‐stratification weighted proportions^b^ of school years 5–11									
F1	92.6	92.0	93.2	5.8	5.4	6.1	1.6	1.4	1.8
F2	93.0	92.4	93.6	4.7	4.4	5.0	2.3	2.1	2.5
F3	94.5	94.0	95.1	4.1	3.8	4.4	1.4	1.2	1.6
Post‐stratification weighted proportions^b^ of school years 7–11									
F1	94.5	94.1	94.8	4.2	4.0	4.4	1.3	1.2	1.4
F2	91.8	91.4	92.3	5.6	5.3	5.8	2.6	2.4	2.8
F3	95.5	95.2	95.8	3.2	3.0	3.4	1.3	1.1	1.4

*Note*: LB and UB refer to the lower and upper bounds of the 99% credible or confidence intervals.

^a^F1–F3 refers to the three food insecurity questions used in Table 1.

^b^ To calculate prevalence using post‐stratification weighting, we used the population of students in state‐funded or independent primary and secondary schools, in national curriculum year groups 5–11 (approx. 9–16 years) in England. This excludes children and young people who are home‐schooled or in alternate provision education settings.

### Research question 2: The association between food insecurity and mental health and wellbeing

**FIGURE 1:**
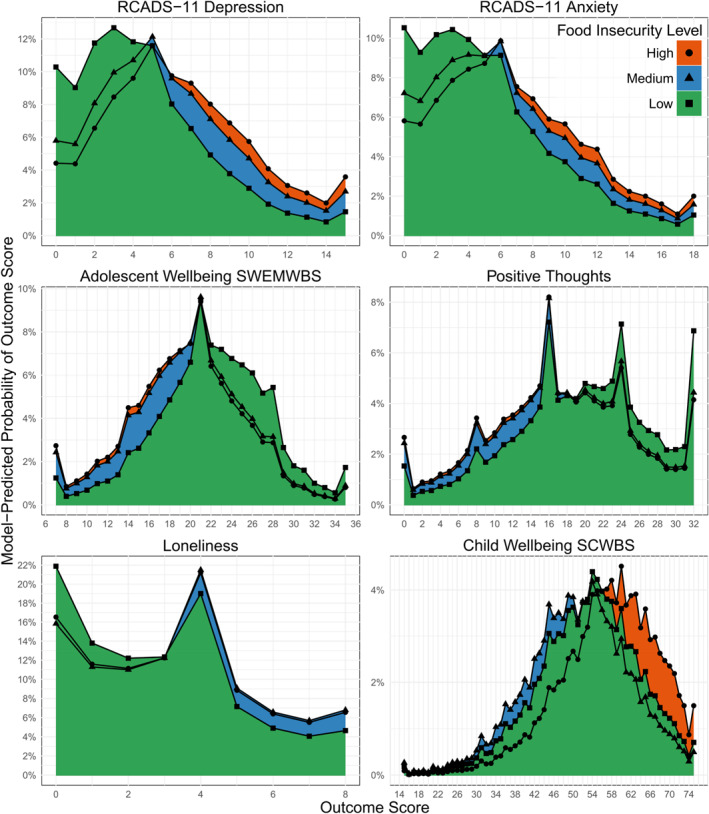
Model‐predicted outcome distributions at low, medium and high food insecurity levels. Each food insecurity level (low, medium and high) represents a hypothetical participant with average scores on all covariates, but who responded *never*, *sometimes* or *often* to all food insecurity questions, respectively. The predicted probabilities are estimated using the fitted ordinal regression models, which capture the overall shape and key features of the original outcome distribution, including floor and ceiling effects and peaks around specific outcome scores, as observed for positive thoughts. The effect of food insecurity is most pronounced for depression scores, with the prediction lines furthest apart across levels. For example, the model predicts that the probability of observing the lowest depression score (0) increases from approximately 4% in the high food insecurity scenario to around 10% in the low food insecurity scenario.

Regression coefficients for each food insecurity question and covariate are reported in the Supplement S8. Each food insecurity level represents a typical participant (i.e., someone average on all covariates) who responded *never, sometimes* or *always* to all three food insecurity questions (see section ‘Methods’). Figure 1 illustrates the model‐predicted distribution of each outcome for low, medium and high food insecurity levels. The model‐predicted mean outcome score for each level of food insecurity is presented in Table 5. Changes in the model‐predicted mean outcome between low and medium levels, as well as between low and high levels of food insecurity, are also presented in Table 5. Changes are reported as raw mean differences on the questionnaire scores, as well as SMD. Note that these estimates control for covariates included in the model.

**TABLE 5:** Marginal effects from ordinal logistic regression models controlling for demographic and deprivation factors.

	Food insecurity level	Model predictions			Comparison from low F.I. level			Standardised mean difference (SMD)			pd (%)
		Est	99% credible interval		Δ	99% credible interval		SMD	99% credible interval		
			LB	UB		LB	UB		LB	UB	
RCADS‐11 depression (whole sample)	Low	4.58	4.47	4.68							
	Medium	5.82	5.43	6.22	1.24	0.85	1.64	0.34	0.24	0.45	100.0
	High	6.43	5.69	7.16	1.86	1.12	2.59	0.51	0.32	0.70	100.0
RCADS‐11 anxiety (whole sample)	Low	5.22	5.10	5.35							
	Medium	6.21	5.74	6.68	0.98	0.52	1.47	0.23	0.12	0.34	100.0
	High	6.79	5.93	7.71	1.57	0.66	2.45	0.36	0.16	0.56	100.0
Adolescent wellbeing SWEMWBS (secondary/FEC only)	Low	22.08	21.90	22.26							
	Medium	19.98	19.27	20.66	−2.10	−2.82	−1.40	−0.37	−0.50	−0.25	100.0
	High	19.62	18.29	20.94	−2.46	−3.80	−1.13	−0.44	−0.67	−0.20	100.0
Positive thoughts (secondary/FEC only)	Low	19.55	19.30	19.80							
	Medium	17.50	16.45	18.55	−2.05	−3.12	−0.97	−0.26	−0.39	−0.12	100.0
	High	17.14	15.12	19.21	−2.41	−4.46	−0.34	−0.30	−0.56	−0.04	99.9
Loneliness (secondary/FEC only)	Low	2.82	2.73	2.91							
	Medium	3.35	3.05	3.66	0.53	0.23	0.84	0.22	0.10	0.35	100.0
	High	3.29	2.75	3.83	0.47	−0.07	1.01	0.20	−0.03	0.42	98.8
Child wellbeing SCWBS (primary only)	Low	53.00	52.44	53.55							
	Medium	50.83	48.35	53.36	−2.17	−4.68	0.39	−0.20	−0.43	0.04	98.6
	High	57.10	53.14	60.90	4.10	0.17	8.01	0.40	−0.00	0.79	99.6

*Note*: Note that sample participants are not classified into different food insecurity levels. Marginal effects are calculated after model fitting and are used to model the expected value of the outcome under different scenarios, depending on the explanatory variables. Each food insecurity level is defined in the statistical analysis section of ‘Methods’. Probability of direction (pd) estimates of pd > 97.5%, pd > 99.5%, pd > 99.95% correspond approximately to *p*‐values of *p* < 0.05, *p* < 0.01 and *p* < 0.001. LB and UB indicate the lower and upper bounds of the 99% credible intervals. The RCADS‐11 was administered to all participants, but other outcomes were presented either to primary or secondary school participants only.

Abbreviation: RCADS, revised children's anxiety and depression scale.

Higher levels of food insecurity were generally associated with decreased mental health and wellbeing.

Relative to low food insecurity, medium food insecurity was associated (pd > 99.5%) with increased model‐predicted RCADS‐11 depression (Δ = 1.24), RCADS‐11 anxiety (Δ = 0.98) and loneliness (Δ = 0.53), and decreased positive thoughts (Δ = −2.05) and adolescent wellbeing (Δ = −2.10). Estimated associations with child wellbeing were unclear in direction (Δ = −2.17, 99% CI [−4.68, 0.39], pd = 98.6%). SMDs comparing low and medium food insecurity ranged from 0.20 to 0.37 in magnitude, considered small to medium effects.

Comparisons of low to high food insecurity levels were generally slightly larger than comparisons of low to medium food insecurity levels (see Table 5). The largest standardised effect size was observed for RCADS‐11 depression scores, where a change from low to high food insecurity was associated with an increase in 0.51 standard deviations (99% CI [0.32, 0.70]), considered a medium effect size. However, changes in the model‐predicted loneliness scores were less certain in direction (pd = 98.8%) than our pre‐registered evidence threshold (pd > 99.5%). In primary school children, we unexpectedly observed a trend towards higher estimated wellbeing in the high food insecurity level. However, this effect size is small and relatively uncertain, only just meeting our evidence threshold (Δ = 4.10, 99% CI [0.17, 8.01], pd = 99.6%). Note that this association is based on a small number of primary school students who responded *Often* to the food insecurity questions (132–200 students).

The ordinal regression coefficients (reported in Supplement S8) indicate the relative association of each food insecurity question with each outcome. *Often* or *sometimes* going to bed hungry was robustly associated with most outcomes in the predicted direction. In contrast, reported food bank usage and food affordability had weaker associations with most outcomes. For predicting wellbeing in primary school children, *often* going to bed hungry was again associated with worse wellbeing, but *Often* responses to the food bank and food affordability questions were associated with slightly better wellbeing. However, it is possible that younger children may have misunderstood these questions.

### Sensitivity analyses

#### Food insecurity and child wellbeing (SCWBS)

Supplement S10 presents descriptive statistics of the Stirling Childhood Wellbeing Scale stratified by food insecurity response. The results show that primary school students who responded *never* or *often* to the first two food insecurity questions (F1–F2) have similar average wellbeing levels, and those who responded *sometimes* had the lowest wellbeing levels. In contrast, for the question about going to bed hungry (F3), child wellbeing scores in the *never* (*M* = 43.6), *sometimes* (*M* = 37.9), and *often* (*M* = 35.8) groups progressively decreased, as hypothesised. When we ran the ordinal regression analyses with no covariates, the comparison of low to medium food insecurity levels was large and negative (SMD = −0.82, 99% CI [−1.04, −0.61], PD = 100%), and the comparison of low to high food insecurity was small, negative and unclear in direction (SMD = −0.22, 99% CI [−0.57, 0.15], PD = 94.5%). These inconsistent findings highlight the need to be cautious when interpreting associations between overall food insecurity level and childhood wellbeing scores in the primary school group.

#### Does removing primary school data alter associations between food insecurity and RCADS‐11 depression and anxiety scores?

Due to concerns regarding whether younger children have misinterpreted the food insecurity questions, we tested whether removing primary school data altered associations between food insecurity and RCADS‐11 depression and anxiety scores. These effects are presented in Table 6. Estimated marginal means and SMD were extremely similar to the results from the whole sample (see Table 5), with three effects slightly decreasing and one slightly increasing.

**TABLE 6:** Reanalysis of RCADS‐11 outcomes without data from primary school students.

Outcome (school groups)	Food insecurity level	Model predictions			Comparison from low F.I. level			Standardised mean difference (SMD)			pd (%)
		*Est*	99% credible interval		Δ	99% credible interval		SMD	99% credible interval		
			LB	UB		LB	UB		LB	UB	
RCADS‐11 depression (secondary/FEC only)	Low	4.87	4.74	4.99							
	Medium	5.98	5.51	6.46	1.11	0.65	1.60	0.30	0.18	0.42	100.0
	High	6.74	5.80	7.69	1.87	0.94	2.83	0.49	0.25	0.73	100.0
RCADS‐11 anxiety (secondary/FEC only)	Low	5.34	5.18	5.50							
	Medium	6.13	5.57	6.70	0.79	0.22	1.35	0.18	0.05	0.31	100.0
	High	7.43	6.30	8.58	2.09	0.96	3.25	0.47	0.22	0.71	100.0

*Note*: Marginal effects are estimated from ordinal logistic regression models controlling for demographic and deprivation factors. Note that sample participants are not classified into different food insecurity levels. Marginal effects are calculated *after* model fitting and are used to model the expected value of the outcome under different scenarios, depending on the explanatory variables. Each food insecurity level is defined in the statistical analysis section of ‘Methods’. Probability of direction (pd) estimates of pd > 97.5%, pd > 99.5%, pd > 99.95% correspond approximately to *p*‐values of *p* < 0.05, *p* < 0.01 and *p* < 0.001. The RCADS‐11 was administered to all participants, but in this table, models were fitted only on secondary school and further education college (FEC) students.

Abbreviation: RCADS, revised children's anxiety and depression scale.

#### Testing for moderation by school year, gender and ethnicity

We explored whether associations between food insecurity and outcomes were moderated by school year, gender and ethnicity (see Supplement S9). Associations varied slightly across demographic groups, but no consistent trends were observed. Due to the small percentage of participants responding *sometimes* or *often* to the food insecurity questions, our estimates within specific demographic groups were highly uncertain, with large, overlapping credible intervals.

## CONCLUSIONS

The cost‐of‐living crisis has brought renewed attention to the issue of childhood poverty and its potentially harmful effects on the mental health and wellbeing of children and young people. In this study, we estimated the prevalence of food insecurity and its association with mental health and wellbeing in a large and diverse sample of children and young people in England in 2023. Participants answered three questions concerning their experiences of food insecurity (family food bank usage, being unable to afford food in school and going to bed hungry) and completed a detailed battery of wellbeing and mental health questionnaires. We found that around 5%–8% of children and young people experienced each dimension of food insecurity either *sometimes* or *often*. Furthermore, higher food insecurity levels were associated with higher depression and anxiety symptoms and reduced adolescent wellbeing and positive thoughts. Food insecurity was weakly related to loneliness and child wellbeing. Out of the three food insecurity questions, going to bed hungry was the strongest predictor of depression and anxiety symptoms.

### Prevalence of food insecurity

Self‐reported food insecurity was highly prevalent among children and young people in our sample, consistent with rates reported in recent studies in England (Armstrong et al., 2023; Leach et al., 2024; UK Government, 2023). Importantly, these estimates only capture the most extreme cases of food insecurity, and might not include households where parents have reduced the quality or variety of food purchased or are otherwise struggling with affordability (Nord, 2013). Indeed, rates of food insecurity derived from parent reports are higher, such as the 12% rate reported in the 2021–2022 Family Resources Survey (UK Government, 2023). Additionally, since data collection occurred during school term time, the provision of free school meals and breakfast clubs may have suppressed food insecurity rates, which would likely be higher during school holidays (Long et al., 2018). Our study only sampled students in four counties in England, which may have different rates of food insecurity relative to other parts of England.

Not many large surveys in the United Kingdom have focused on households with children and young people, and our findings highlight some novel trends (see Supplement S5). Participants identifying as male reported slightly higher rates of going to bed hungry and food bank usage than those who identified as female. This may reflect higher average energy intake requirements in males (Committee on the Dietary Reference Intakes for Energy et al., 2023), requiring increased food expenditure. In our sample, gender‐diverse students reported higher food insecurity than females or males (see Supplement S5). One Finnish study of adolescents found that higher socioeconomic status correlates with cisgender identity, potentially explaining part of this effect (Kaltiala et al., 2023). Another recent study also found higher rates of food insecurity in transgender and gender nonconforming adolescents in Canada (Hallward et al., 2023). We also observed higher rates of food insecurity in Black and Other (including Arab) ethnic groups, consistent with findings from previous surveys, potentially reflecting socioeconomic differences between groups (Harris et al., 2023).

Exploratory analyses indicated that the prevalence of food insecurity varied by school year group, although trends were non‐linear and varied per question (see Supplement S6). Notably, the proportion of students reporting being unable to eat sometimes or often at school nearly doubled between primary school years 5–6 (4.1%, *N* = 8518) and secondary school years 7–8 (8.16%, *N* = 11,795). This might reflect differences in the costs or provision of school meals between primary and secondary school. However, this could be an artefact of primary school children interpreting the questions differently. More research is needed to understand this aspect of food insecurity in children and young people.

### Associations with mental health problems

We modelled associations between food insecurity and six mental health and wellbeing outcome variables using Bayesian ordinal regression models, controlling gender, school year, ethnicity, birth location, parental birth location, deprivation and education setting. As predicted, increased food insecurity was associated with elevated depression and anxiety scores, and reduced adolescent wellbeing and positive thoughts. However, associations with loneliness and child wellbeing were small and unclear in direction.

Several mechanisms might link food insecurity to wellbeing and mental health. Experimental research suggests that acute food restriction can decrease mood (Ackermans et al., 2022). Food insecurity may also influence wellbeing through anxiety about available food and parental stress (Fram et al., 2011; Leung et al., 2020). Qualitative research indicates that children and young people in food insecure households report worrying about available food, and that some report embarrassment about their household circumstances, which may disrupt social relationships with peers not facing similar challenges (Leung et al., 2020). Relative deprivation theories highlight the role of social comparisons in determining self‐perceptions of our circumstances (Piera Pi‐Sunyer et al., 2023). Indeed, one study found that the effect of food insecurity on wellbeing is stronger in countries with less overall food insecurity (Elgar et al., 2021). The effect of food insecurity may be stronger for those living in affluent neighbourhoods or schools, where more deprived children and young people might experience this deprivation more acutely or become isolated if they feel they cannot spend time with their peers as a result. Because we controlled for deprivation, the small association between loneliness and food insecurity could be explained if loneliness is associated with general deprivation, but not food insecurity specifically.

Going to bed hungry due to a lack of food had the largest and most consistent associations with the outcome variables. This question is potentially a clearer measure of severe food insecurity and less open to misinterpretation—though it likely only captures extreme cases. The experience of going to bed hungry may be particularly distressing and could disrupt sleep, which is linked with overall wellbeing and development (Nagata et al., 2019).

Unexpectedly, we found a trend of higher Stirling Childhood Wellbeing Scores in the high food insecurity level relative to the low food insecurity level. However, this finding should not be considered robust or conclusive. Firstly, this effect only marginally passed our evidence threshold (pd > 99.5%), so it could represent a false positive finding. Second, this effect reversed direction when not controlling for covariates. Third, inspection of the regression coefficients reveals that *often* responses to the three food insecurity questions have inconsistent (some positive, some negative) associations with the outcome, whereas *sometimes* responses were consistent and in the expected direction. It is possible that some primary school children misunderstood questions 1 and 2, for example, by not knowing what a food bank was. This potential misunderstanding could have led to the inconsistent results observed in the high food insecurity group. Caution should be exercised when interpreting the relationship between food insecurity and wellbeing scores in primary school children, particularly for those who reported frequent food insecurity experiences.

Multivariable regression coefficients for demographic factors associated with mental health outcomes reveal several noteworthy trends, which are reported in full in Section 8 of Supporting Information S1. Gender was associated with all mental health outcomes, with males generally reporting better outcomes relative to females on average, while gender‐diverse individuals reported worse outcomes than females or males. These results align with recent research in the UK indicating increased mental health problems among female and gender‐diverse adolescents (Anthony et al., 2024; Armitage et al., 2023; Price‐Feeney et al., 2020). Relative to White ethnicity, Asian and Black ethnic groups reported slightly lower depression and anxiety scores, along with slightly higher wellbeing (SWEMWBS) and positive thoughts. Loneliness scores were slightly lower among Asian and other ethnic groups relative to White participants. This finding aligns with recent results from the #BeeWell study of 35,000 adolescents in Greater Manchester, which also found slightly lower loneliness scores in Black and Asian adolescents relative to White adolescents (Marquez et al., 2023). Speculatively, this difference in loneliness may reflect a stronger role of family connections in social relationships within some ethnic minority communities (Miles et al., 2012; Stein et al., 2015). However, most differences we found between ethnic groups were small, with SMDs ranging from 0.06 to 0.22. Being born in the UK or having a parent born in the UK was not robustly associated with any outcomes in the regression analyses (pd < 99.5%).

### Limitations

This study has several limitations. Due to the observational design, the modelled associations between food insecurity and outcomes may not reflect true causal effects. An aspect of the OxWell survey design is that no identifiable data are collected, in the hope that participants will feel able to answer the questions accurately. However, because participants are not linkable to household or neighbourhood data, this limits the collection of additional variables that would aid statistical control or weighting. We controlled for deprivation using self‐reported deprivation questions, which are not perfect measures of family income (Torsheim et al., 2016). This means that residual confounding by household income is possible, and we cannot fully separate the effect of food insecurity from other dimensions of poverty.

Another limitation of this study is the use of only three questions to measure food insecurity, which may be less reliable than a longer, validated scale (e.g., Revelle & Condon, 2018). However, the strong polychoric correlations between the three items (ranging from 0.53 to 0.68) indicate that they likely capture a consistent underlying construct. One potential concern is whether younger students, particularly in primary school, fully understood all the food insecurity questions. One question (F3: ‘At home, I go to bed hungry because there is not enough food in the house’) emerged as a considerably stronger predictor of adverse outcomes compared to the other two questions. This finding suggests that this specific item might be more effective in identifying severe cases of food insecurity or could be less susceptible to misinterpretation.

Finally, although our sample is large and diverse, we cannot fully assess its representativeness. The anonymous data collection design, while protecting participant privacy, prevents us from calculating school‐level participation rates or examining potential selection biases. Our sampling frame is limited in two important ways. First, the survey only covers four regions of England, excludes students in special or alternative provision schools (1.9% of the school population in 2023/2024; Department for Education, 2024b) and those who are home‐educated (1.1% of students in 2023/2024 Autumn; Department for Education, 2024a). Second, the survey only includes children and young people present in school on the day of testing. This may underestimate prevalence given that socioeconomic disadvantage is slightly associated with lower school attendance (Klein et al., 2020; Sosu et al., 2021). In addition, estimates of the association between food insecurity and mental health may be subject to collider bias if both factors influence the probability of school absence, and hence inclusion in the study (Hernán & Monge, 2023; Heyne et al., 2022).

### Conclusion

Our findings highlight the concerning rates of food insecurity among children and young people in England. These results emphasise the potential negative consequences of severe economic deprivation and highlight the importance of identifying children and young people at risk of food insecurity and the potential mental health consequences, including depression.

## AUTHOR CONTRIBUTIONS

**Giacomo Bignardi**: Conceptualization; methodology; software; data curation; visualization; writing—original draft. **Mina Fazel**: Conceptualization; methodology; data curation; funding acquisition; writing—review and editing. **Sarah‐Jayne Blakemore**: Conceptualization; methodology; supervision; funding acquisition; writing—review and editing.

## CONFLICT OF INTEREST STATEMENT

The authors declare no conflicts of interest.

## ETHICAL CONSIDERATIONS

The study was approved by the University of Oxford Medical Sciences Interdivisional Research Ethics Committee (Reference: R62366/RE014) on 19 January 2023. Students were enroled using a parental opt‐out model along with students' assent (student age <16 years) or students' informed consent only (student age ≥16 years).

## Supporting information

Supplementary Material S1

## Data Availability

G.B. and M.F. had full access to all the data in the study and accept responsibility to submit for publication. The data are not publicly available due to ethical and information governance restrictions. The data that support the findings are available upon reasonable request. Researchers may access the data by applying through the BrainWaves Data Portal (https://brainwaveshub.org/for‐research/) where applications are reviewed to ensure appropriate use. Further details, including the full list of questions, study protocol, and other supporting materials, are available via the OxWell project's Open Science Framework page: https://osf.io/sekhr/. The analysis code is available at the following link: https://github.com/giac01/4_oxwell_foodinsecurity.
