# Supplementary material for: The prevalence of food insecurity and its relationship with wellbeing in a large, cross‐sectional study of children and young people in England
Source: JCPP Adv. 2025 Sep 23;6(2):e70049. doi: 10.1002/jcv2.70049 (PMC13260701; doi:10.1002/jcv2.70049)
Supplement: Supplementary file 1 — Supplementary Material S1 [file JCV2-6-e70049-s001.docx]

# Supplementary Materials

Supplement S1) Additional descriptive tables

Supplement S2) Missing data descriptive statistics

Supplement S3) Deviations from the pre-registration

Supplement S4) Research question 1: calculation of weights

Supplement S5) Research question 1: predictors of food insecurity

Supplement S6) Research question 1: distribution of food insecurity responses for each school year group

Supplement S7) Research question 2: description of the ordinal regression model

Supplement S8) Research question 2: ordinal regression full results table

Supplement S9) Research question 2: exploratory subgroup analyses using mixed models

Supplement S10) Research question 2: distribution of each outcome variable stratified by food insecurity response

## Supplement S1) Additional descriptive tables

Polychoric Correlations between Food Insecurity Variables

| Food Security Question | 1 | 2 | 3 |
| --- | --- | --- | --- |
| 1) My family uses food banks | 37273 | 37065 | 36990 |
| 2) At school, I am unable to afford to eat | **0·53** | 37609 | 37382 |
| 3) At home, I go to bed hungry because there is not enough food in the house | **0·61** | **0·68** | 37526 |
| **Lower Diagonal Entries**: pairwise polychoric correlations estimated using the function psych::polychoric(., global = FALSE) from the psych R package (v2·3·6)  **Diagonal Entries**: Number of complete (non-missing) observations for each variable  **Upper Diagonal Entries:** Sample size for each pairwise correlation | | | |

Distribution of ages in the eligible sample (Total N = 38,430)

| **Age** | **Count** | **Percentage** |
| --- | --- | --- |
| Missing | 692 | 1.80% |
| 8 | 8 | 0.02% |
| 9 | 1721 | 4.48% |
| 10 | 4287 | 11.16% |
| 11 | 5038 | 13.11% |
| 12 | 5945 | 15.47% |
| 13 | 5720 | 14.88% |
| 14 | 4841 | 12.60% |
| 15 | 4004 | 10.42% |
| 16 | 3309 | 8.61% |
| 17 | 1832 | 4.77% |
| 18 | 825 | 2.15% |
| 19 | 208 | 0.54% |

**Distribution of ethnicity in OxWell and DfE School Census**

| **Ethnicity** | **Sample Totals** | **Sample %** | **Census %** | **Diff** | **Census Totals** |
| --- | --- | --- | --- | --- | --- |
| White | 15753 | 65.2% | 70.3% | -5.1% | 2526856 |
| Asian | 4139 | 17.1% | 13.6% | 3.5% | 489150 |
| Black | 1417 | 5.9% | 6.8% | -0.9% | 244882 |
| Mixed | 1654 | 6.8% | 6.9% | 0.0% | 247020 |
| Other | 1199 | 5.0% | 2.4% | 2.5% | 87035 |
| Missing | 5572 |  |  |  | 74990 |
| Sample data comes from all students in school years 7 – 13 in state secondary schools, further education colleges and independent schools. Census data are from the DfE 2023/2024 school census which only covers state-funded secondary schools and *does not include independent schools.*  DfE census data can found at: <https://explore-education-statistics.service.gov.uk/data-tables/permalink/76c7f698-d38b-42d1-9fea-08dcedcd278c> | | | | | |

The independent schools council 2024 census only reports the overall number of students in “white British” and “UK minority ethnic” groups, and is not broken down by school year, and is therefore not used here.

## Supplement S2) Missing data descriptive statistics

Below, we present pairwise missing plots, which illustrate the number of complete observations for each pair of variables. We present separate plots for primary and secondary schools.

The number on the diagonal indicates the number of complete responses on each item, divided by 100. The numbers below the diagonal indicate the number of participants with complete responses on a pair of items.

For example, the secondary school plot indicates there are ~28,500 non-missing responses for gender, 24,200 non-missing responses for ethnicity, and 23,200 participants have non-missing gender *and* ethnicity responses. By design, in the primary school sample, no students completed questions from the SWEMWBS, positive thoughts (pt). Note that participants in primary school completed one loneliness item (X1780_lone) but not the others.


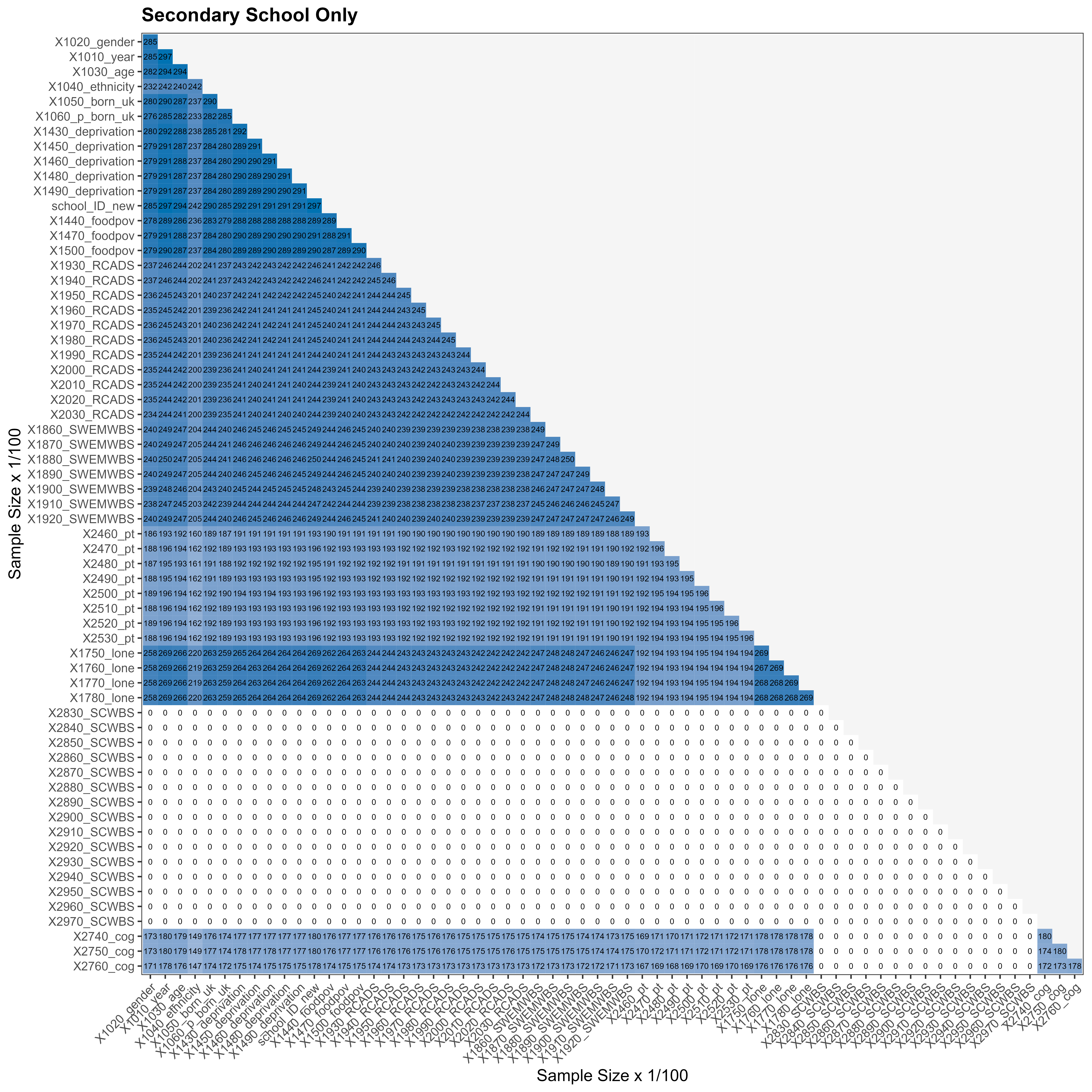


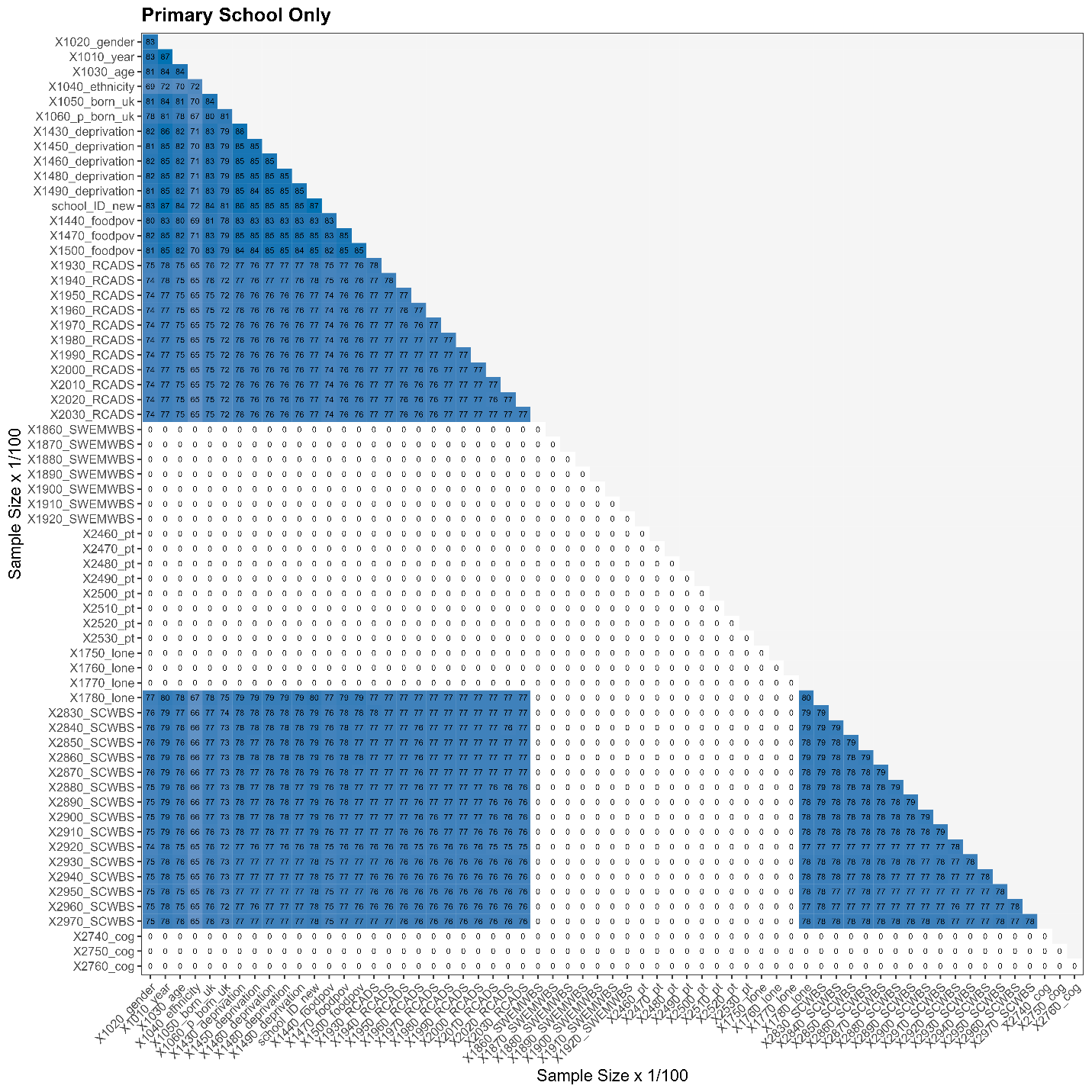


*Note.* Code to create these plots is available from GB [here](https://github.com/giac01/gbtoolbox/blob/main/R/plot_pairwise_missing.R).

## Supplement S3) Deviations from the pre-registration

We made the following changes to our pre-registered analysis plan. The pre-registration can be found at the following site: <https://osf.io/7tdkv>.

**Both Questions**

1. Initially, we planned to use the “other” response category to the gender question. However, inspecting the free text responses to the subsequent “Prefer to self-identify (please describe)” question suggested that several participants gave untruthful responses. The free-text responses were used to filter out these participants as described in the main text.

**Research Question 1**

1. We originally planned to calculate post-stratification weights based on type of school (independent vs other), index of multiple deprivation, if the parent or child is born in the UK, ethnicity, and index of multiple deprivation. Much of this information is missing in the spring 2021 school census, and so weights were only created based on school year and school type. Data from students in school years 12-13 was also omitted due to incomplete census information for these school years. We also used the survey package to calculate weighted prevalence and case count rather than the Bayesian approach outlined in the pre-registration.
2. We added a calculation of the total population cases of food insecurity.
3. In response to reviewer concerns about whether younger children fully understood the food insecurity questions, weighted prevalence estimates are presented both with and without primary school data.

**Research Question 2**

1. Posterior predictive checks indicated that normal distribution regression models poorly captured the overall shape of most outcome variables. Many outcomes had large ceiling and floor effects. Therefore, we opted to use an ordinal regression model instead of a normal distribution model.
2. We increased the number of imputations used to 100, relative to the pre-registered plan.
3. Sensitivity analyses were added that were not pre-registered.

## Supplement S4) Research question 1: calculation of weights

To calculate population totals, we combined data from the DfE 2023/2024 school census and the [2024 Independent Schools Council (ISC) census](https://www.isc.co.uk/media/uukn4r3i/isc_census_2024_15may24.pdf).

**Calculating Census Counts for English Independent Schools**

The DfE census collects data on the number of students in each national curriculum year group in state-funded schools. For independent (private) schools, this data is not broken down by year group. The ICS 2024 census reports the number of students in each year group, however, the census only covers independent schools that are members of constituent associations of the ISC, and includes some schools not in England. 92.1% of students in the ISC 2024 census are located in English schools.

To estimate the headcount of students in English independent schools in each year group, we first calculated the ratio between the total number of students reported in the ISC 2024 census (556,551) to the DfE census number for the total number of students in English independent schools (593,486). There are ×1.06636 more students reported in the DfE census than in the ISC census. We then multiplied this ratio (1.06636) by the number of students in each year group in independent schools (see Table 3, page 31, of the ISC 2024 census report).

**Post-stratified Weights**

In our sample, we included only students in state-funded primary and secondary schools and independent schools in national curriculum year groups 5 – 11 (primarily covering ages 9 – 16 years).

For each food insecurity question (F1, F2, F3; see Table 1 for description), we calculated the number of eligible responders in each year group and school type (state or independent), reported in the table below. Weights were calculated by dividing the number of students in the population (“Census Totals” column) by the sample totals for each cell. Weights were calculated separately for each question to account for different numbers of missing data. Imputation of missing data was not performed.

Weights are calculated by dividing the census cell total by the sample cell total.

Population case counts were calculated using the survey::svytotal() R function. Prevalence estimates were calculated by dividing case counts by the population size.

**Weights, population and sample totals for each post-stratified group**

| School Type | Sch.  Year | Census  Totals | Sample Totals | | | Weights | | |
| --- | --- | --- | --- | --- | --- | --- | --- | --- |
|  |  |  | F1  Responders | F2  Responders | F3  Responders | F1 | F2 | F3 |
| State-funded primary + State-funded secondary | 5 | 642194 | 4097 | 4201 | 4174 | 157 | 153 | 154 |
| State-funded primary + State-funded secondary | 6 | 653044 | 4124 | 4205 | 4201 | 158 | 155 | 155 |
| State-funded primary + State-funded secondary | 7 | 648920 | 5977 | 6062 | 6059 | 109 | 107 | 107 |
| State-funded primary + State-funded secondary | 8 | 643836 | 5475 | 5516 | 5501 | 118 | 117 | 117 |
| State-funded primary + State-funded secondary | 9 | 629079 | 5057 | 5071 | 5077 | 124 | 124 | 124 |
| State-funded primary + State-funded secondary | 10 | 617557 | 3808 | 3813 | 3804 | 162 | 162 | 162 |
| State-funded primary + State-funded secondary | 11 | 614522 | 3650 | 3658 | 3644 | 168 | 168 | 169 |
| Independent | 5 | 33264 | 58 | 58 | 58 | 574 | 574 | 574 |
| Independent | 6 | 36273 | 55 | 54 | 54 | 660 | 672 | 672 |
| Independent | 7 | 48259 | 59 | 59 | 59 | 818 | 818 | 818 |
| Independent | 8 | 50878 | 157 | 158 | 159 | 324 | 322 | 320 |
| Independent | 9 | 53410 | 291 | 289 | 291 | 184 | 185 | 184 |
| Independent | 10 | 55238 | 273 | 274 | 274 | 202 | 202 | 202 |
| Independent | 11 | 57111 | 183 | 181 | 182 | 312 | 316 | 314 |
| Totals |  | 4,783,586 | 33,264 | 33,599 | 33,537 |  |  |  |
| Note. Our population estimates only include students in state-funded primary and secondary schools in year groups 5 –11 in England. Independent school census totals are inferred from DfE and ISC 2024 census data. | | | | | | | | |

**Limitations**

Our approach assumes that the year group distribution of students in English independent schools is similar to the UK-wide distribution reported in the ISC census. Our estimates also rely on the DfE school census estimates of the number of students in independent schools. We did not use gender weights because OxWell measured self-reported gender identity and the DfE census measures sex. We could not identify DfE census data which cross-tabulated ethnicity and year group.

## Supplement S5) Research question 1: predictors of food insecurity

We explored the predictors of food insecurity using ordinal logistic regression. The results of these analyses are presented below.

Each food insecurity question was analysed separately and entered as the outcome variable of the model. Because there are three possible response options (never, sometimes, often), the model estimates two intercepts (or “thresholds”). Explanatory variables are entered into the model using dummy coding. School ID is entered into the model as a random intercept. Models were fitted using the same rstan ordinal regression models used in the second research question analyses.

Regression coefficients from the model are presented below, along with the posterior means (Est) and 99% highest density credible intervals. We report the probability of direction (PD) for each coefficient. All regression coefficients were divided by the standard deviation of the standard logistic distribution $(\pi\times3^{-0.5})$, so that they could be interpreted as standardised mean differences comparing food insecurity between the reference and dummy coded groups.

Children and young people identifying as male were slightly more likely to report family food bank usage (SMD = .104, pd = 100%) and going to bed hungry (SMD = .152, pd = 100%), but were not much more likely to report being able to afford to eat in school (SMD = .029, pd = 89.3%). Rates of food insecurity were broadly similar across White, Mixed and Asian Ethnicity groups, and elevated for Black ethnicity students (SMD between .166-.209). Students not born in the UK were more likely to report food bank usage (SMD = .188, pd = 100%) and not being able to afford to eat in school (SMD = .151, pd = 100%), but only a small difference in going to bed hungry (SMD = .074, pd = 96%).

Relative to children in school year 9 (ages 13 – 14), children in school years 5 (ages 9 – 10) and 6 (ages 10 – 11) were much more likely to report food bank usage (SMD = .626 and .506, respectively) and to go to bed hungry (SMD = .501 and .255), but less likely to report being unable to afford to eat in school (SMD = -.235 and -.538). Young people in school years 12 (ages 16 – 17) and 13 (ages 17 – 18) reported slightly higher food insecurity rates than year 9 students. Food insecurity rates differed between schools, as the random intercepts had a non-zero standard deviation (SD = .41 – .49).


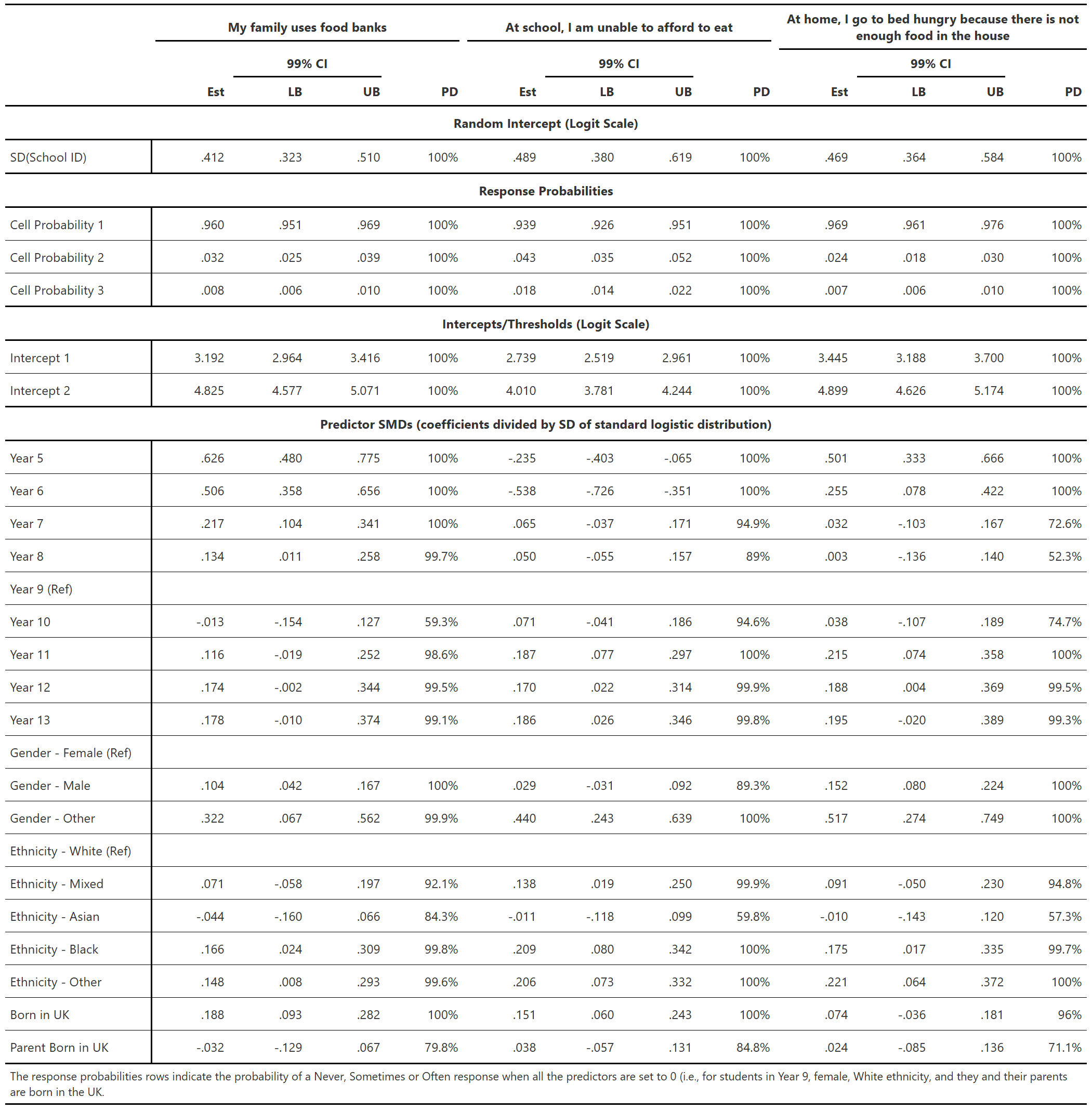


## Supplement S6) Research question 1: distribution of food insecurity responses for each school year group

Below, we present the number of *Often* and *Sometimes* responses to each food insecurity question, split by year group. The total number of *Never, Often* and *Sometimes* responses is also presented (“Total N”).


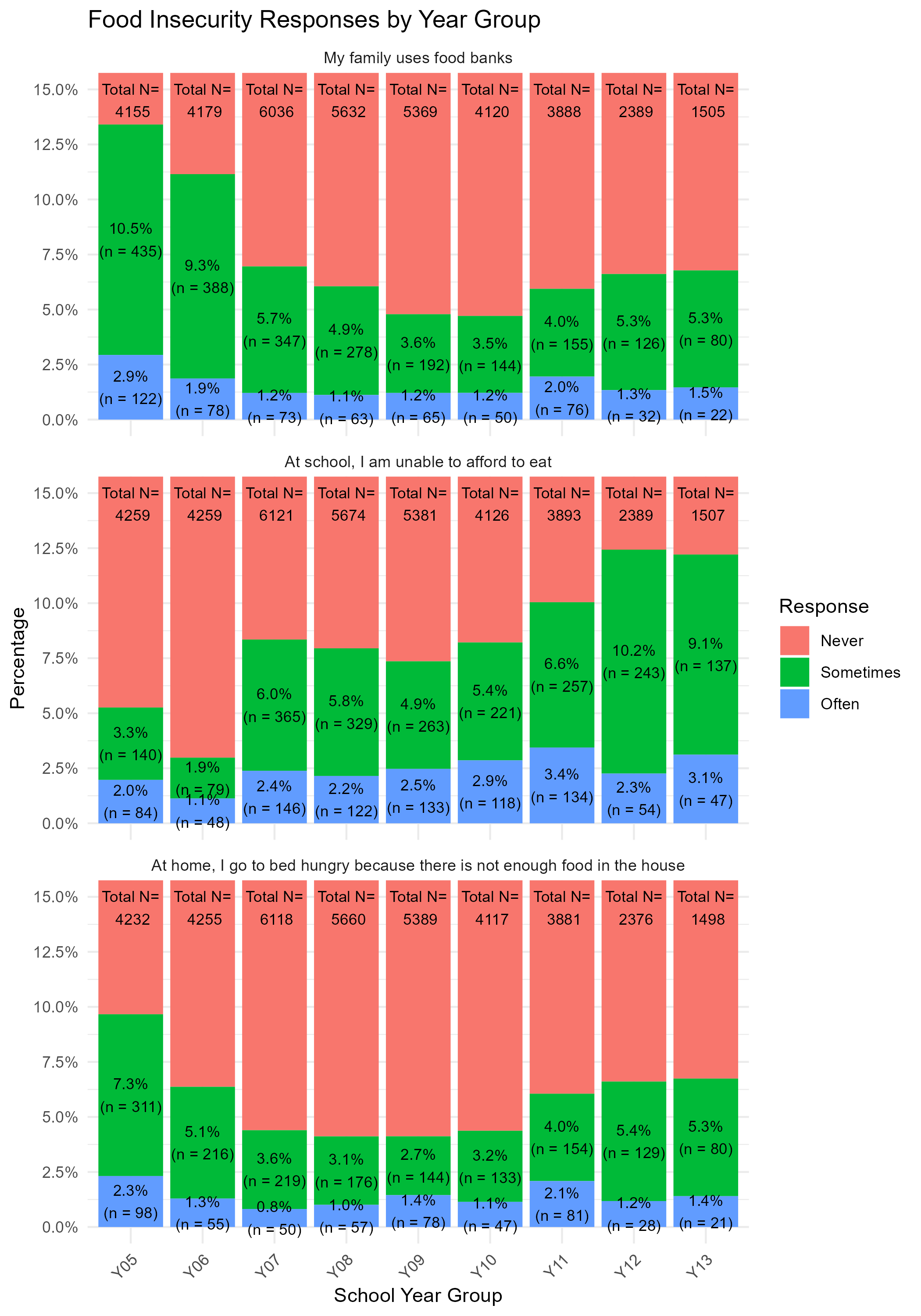


## Supplement S7) Research question 2: description of the ordinal regression model

This section begins with a detailed verbal description of the model components and concludes with a concise mathematical overview of the model.

**Background to Ordinal Logistic Regression**

Ordinal logistic regression models were used to model each outcome variable. This model assumes that participants' ordinal outcome responses (e.g., depression scores) are caused by a continuous latent variable (e.g., latent depression score). Latent depression scores within certain boundaries lead to the same observed ordinal response level. These boundaries are called the **cutpoints**. In the example below, the observed ordinal variable has 5 response levels (K = 5). Participants with a latent score between the second and third cutpoints would respond “3” on the ordinal scale, and participants with a latent score above the 4^th^ cutpoint would answer “5”. For outcomes with K ordinal levels, K–1 cutpoints are needed. The cutpoints and latent scores are estimated within the model and not directly observed.


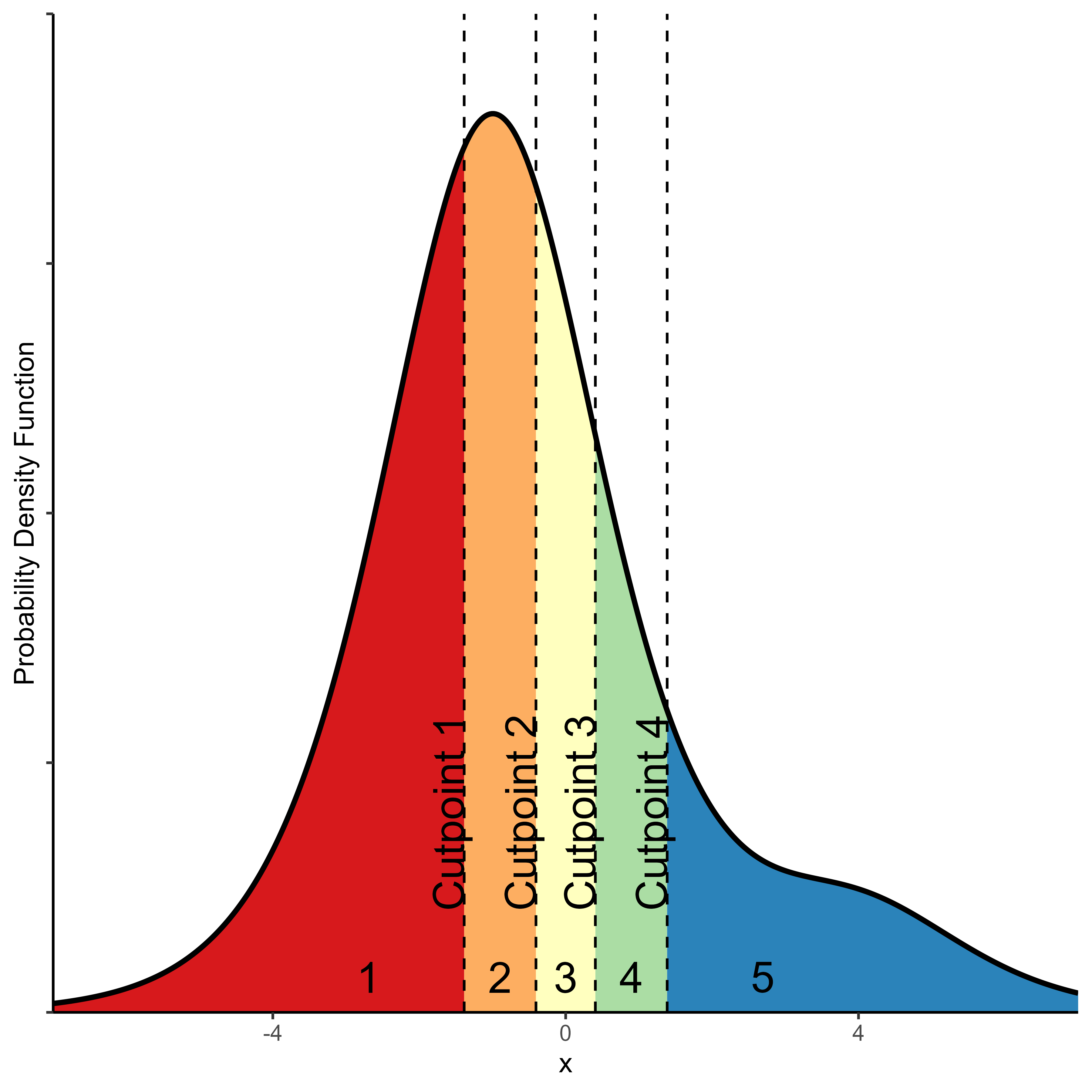


The proportion of observed, ordinal responses in each level is determined by the shape of the latent outcome distribution and the location of the cutpoints. The area of each segment above determines the probability of each ordinal outcome. If we randomly draw participants from the latent distribution observed above, we expect that 36% of the sample would respond “1” and 16% would respond “5”.

Some restrictions are imposed on the shape of the continuous latent outcome for model identification. In ordinal logistic regression, we assume the latent outcome variable follows a conditional logistic distribution. The location of this distribution can shift to the left or right, depending on the explanatory variables, via the linear predictor term.

Like standard regression, the latent, continuous outcome variable is regressed onto the explanatory variables. The explanatory variables are linked to the latent outcome using a linear combination of regression coefficients $(\beta$) and explanatory variables. The key difference here is that the outcome ($\tilde{Y}_{i}$) is latent, and the error term follows a standard logistic distribution. The standard logistic distribution has location and scale parameters of 0 and 1, corresponding to a mean and standard deviation of 0 and ~1.81, respectively. For a set of *P* explanatory variables, the formula predicting the latent outcome from the explanatory variables looks like the following:

$$\tilde{Y}_{i}=\boldsymbol{\eta}_{\boldsymbol{i}}+ \varepsilon= \beta_{1}x_{1i}+\beta_{2}x_{2i}+\ldots+ \beta_{P}x_{Pi}+ \varepsilon_{i}$$

$$\varepsilon_{i}\sim Logistic Distribution \left( location= 0, scale=1 \right)$$

When we have no explanatory variables, the linear predictor term is removed $(\eta_{i}=0)$, and the above equation reduces to $\tilde{Y}_{i}= \varepsilon,$ and the latent scores follow a standard logistic distribution. When explanatory variables are included in the model, and the regression coefficients are non-zero, the explanatory variables can shift the distribution of the latent outcome to the left or right. The mean of the latent scores $\left( E\left[ \tilde{Y} \right] \right)$ is determined by the linear predictor term ($\eta_{i})$:

$$E\left[ \tilde{Y} \right]= \eta_{i}+E\left[ \varepsilon_{i} \right]=\eta_{i}+0= \eta_{i}$$

Let’s say we have a single explanatory variable that is dummy-coded so that $x_{1i}=0$ when participant *i* is in group 1, and $x_{1i}=1$ when participant *i* is in group 2. The beta coefficient for the explanatory variable ($\beta_{1})$ now determines the degree of separation between the latent outcome scores of the two groups:

$$\tilde{Y}_{i}=\beta_{1}x_{1i}+ \varepsilon$$

$$When x_{1i}=0, \tilde{Y}_{i}= \varepsilon$$

$$When x_{1i}=1, \tilde{Y}_{i}=\beta_{1}+ \varepsilon$$

For example, if $\beta_{1}=1.81$, the distribution of the latent outcome in the second group will have the same logistic shape but will be shifted up by 1.81. In the example below, we expect that the percentage of participants responding “5” on the outcome will be 20% and 60% in groups 1 and 2, respectively.

**Distribution of latent and observed scores in groups 1 and 2**


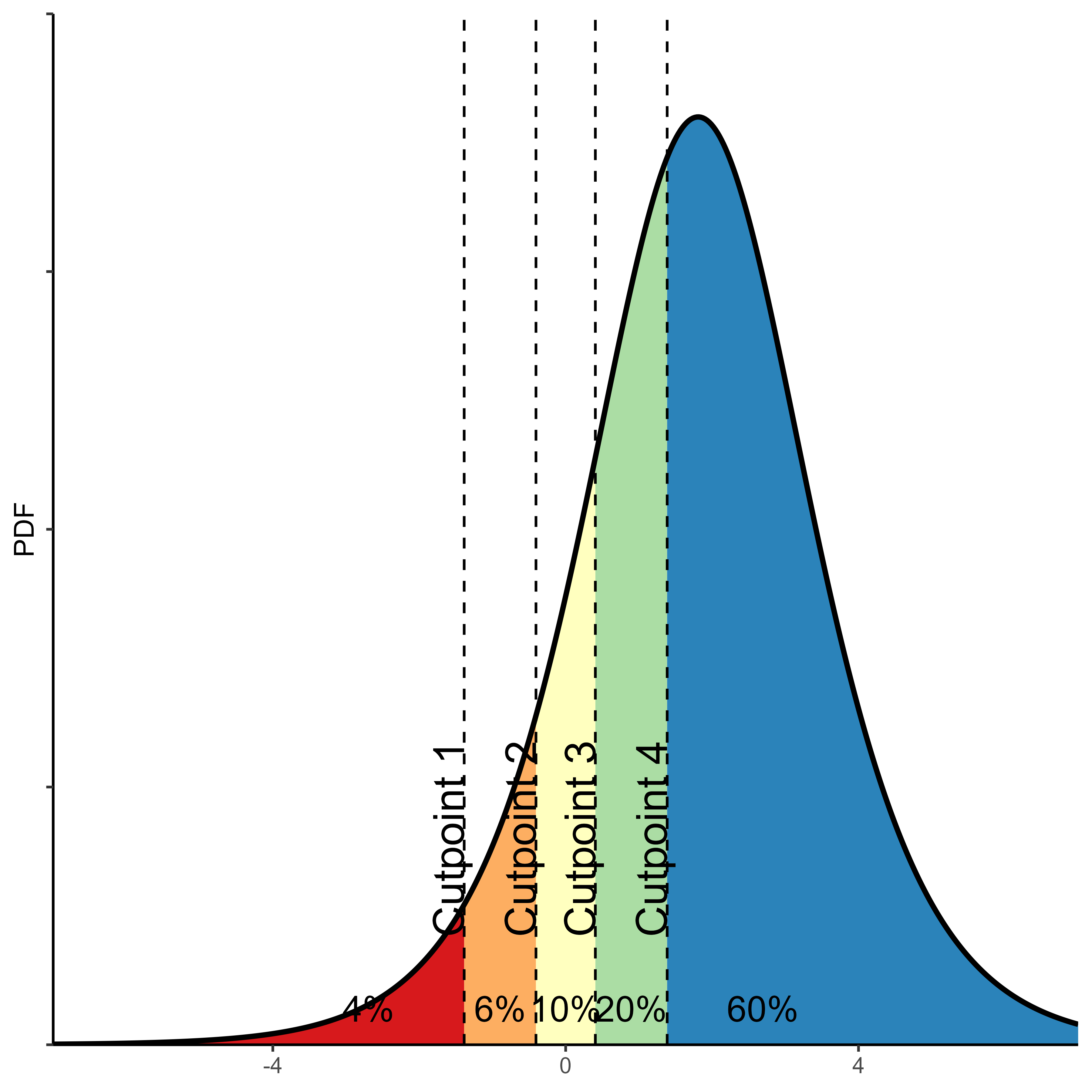

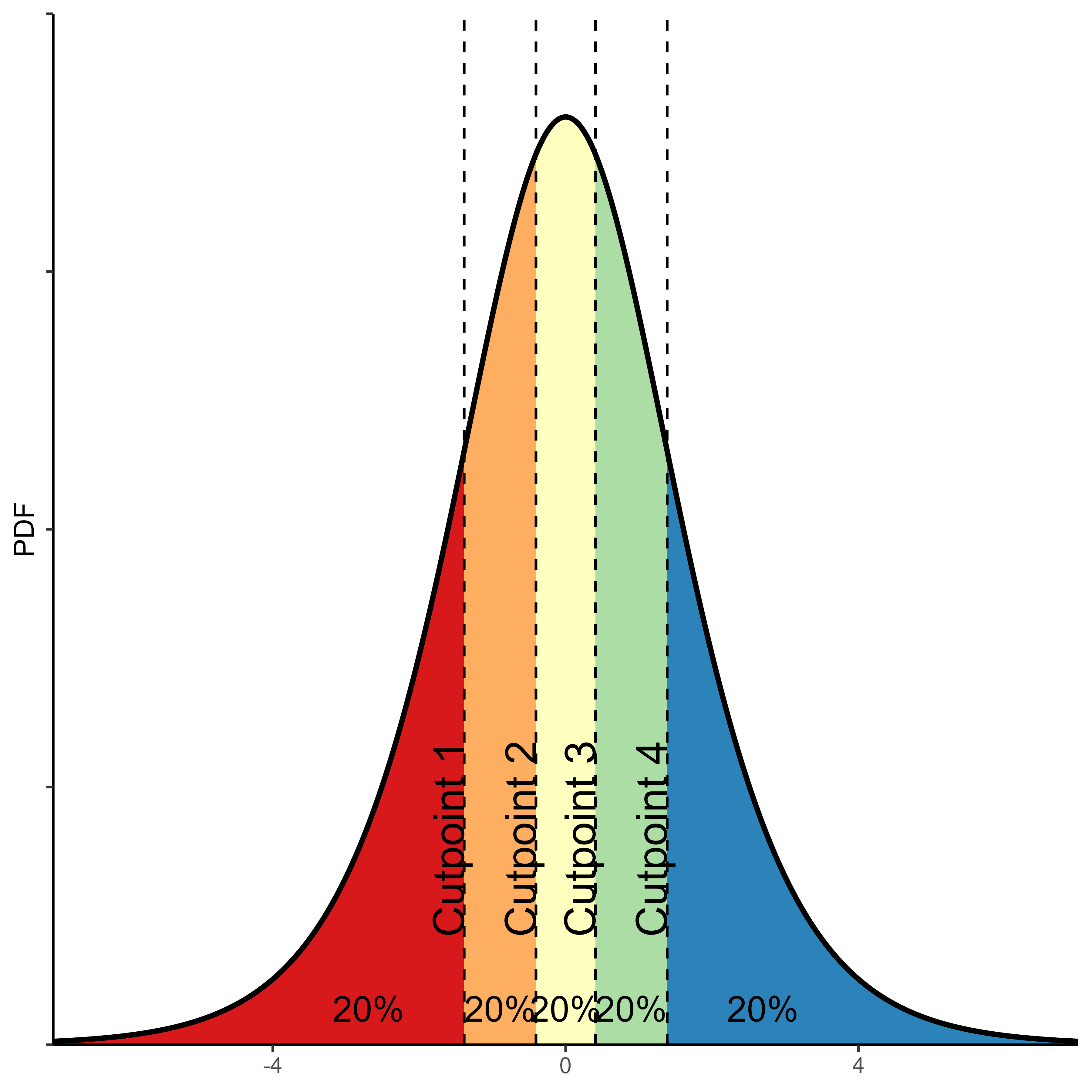


**Group 2** $(\boldsymbol{\eta}_{\boldsymbol{i}}=1.81)$

**Group 1** $(\boldsymbol{\eta}_{\boldsymbol{i}}=0)$

**Group 2 (**$\boldsymbol{\eta}=1.82)$

**Group 1 (**$\boldsymbol{\eta}=0)$

**Estimated Marginal Means (EMMs)**

EMMs are the regression model’s estimate of the mean (or “prediction”) of the outcome at specific values of the predictor variables, defined by a “reference grid”. We calculated EMMs at three levels of food insecurity: low, medium, and high. At each level of this reference grid, the values of the covariates were held at their means. Low food insecurity was defined as responding “never” across all questions, medium food insecurity was defined as responding “sometimes” to each question, and high food insecurity was defined as responding “often” to each question. These three levels represent three hypothetical participants who are “average” on the covariates but have different food insecurity responses.

An example reference grid is shown below. The values of the covariates shown are illustrative only.

| Food Insecurity Level | Covariates  (Held Constant) | | | | | | | | | | | | | Food Insecurity Variables | | | | | |
| --- | --- | --- | --- | --- | --- | --- | --- | --- | --- | --- | --- | --- | --- | --- | --- | --- | --- | --- | --- |
|  | Gender - Male | Gender -Other | X1430 - Often | X1430 - Sometimes | X1450 - Often | X1450 - Sometimes | X1460 - Often | X1460 - Some | X1480 - Often | X1480 - Sometimes | X1490 - Often | X1490 - Sometimes | … | Food Bank - Sometimes | Food Bank - Often | Unable to afford to eat - Sometimes | Unable to afford to eat - Often | Go to bed hungry - Sometimes | Go to bed hungry - Often |
| Low | ·48 | ·02 | ·07 | ·24 | ·02 | ·08 | ·03 | ·07 | ·04 | ·10 | ·02 | ·07 |  | 0 | 0 | 0 | 0 | 0 | 0 |
| Medium | ·48 | ·02 | ·07 | ·24 | ·02 | ·08 | ·03 | ·07 | ·04 | ·10 | ·02 | ·07 |  | 1 | 0 | 1 | 0 | 1 | 0 |
| High | ·48 | ·02 | ·07 | ·24 | ·02 | ·08 | ·03 | ·07 | ·04 | ·10 | ·02 | ·07 |  | 0 | 1 | 0 | 1 | 0 | 1 |

We employed the following procedure to calculate the EMM for a specific level of food insecurity. For each MCMC draw of the posterior distribution, we calculate the linear predictor term $(\eta_{s}=\sum_{p=1}^{P} B_{p,s}x_{p})$, where $x_{p}$ refers to values of the explanatory variables in the reference grid for the relevant food insecurity level. Using the MCMC draws of the linear predictor term $\left( \eta_{s} \right)$ and model cutpoints, we then calculated the predicted probability of each ordinal outcome response level. Finally, the EMM for each draw is calculated by taking a weighted sum of the outcome response levels weighted by their model-predicted probability. The mean and highest continuous density credible interval are then computed using the R function ggdist::mean_hdci.

**Standardised Mean Differences (SMD)**

Standardised mean differences were estimated by modelling change in the latent outcome at different food insecurity levels. For each food insecurity group (i.e., low, medium or high), the distribution of the latent outcome follows a logistic distribution that is shifted to the left or right depending on the linear predictor term $\left( \eta_{s} \right)$. In the example above, the distribution of latent depression scores follows a logistic distribution with a mean of 0 for group 1, and a mean of 1.81 in group 2. The means are determined by the linear predictor term $(\eta_{s}=\sum_{p=1}^{P} B_{p,s}x_{p})$. To calculate the latent mean difference for a given *s* MCMC draw $\left( \tilde{\Delta}_{s} \right)$, for any two groups in our reference grid, we calculate the linear predictor for each group, and then take the difference:

$$\tilde{\Delta}_{s}= \eta_{s,group2}-\eta_{s,group1}=\sum_{p=1}^{P} B_{p,s}x_{p,group2}-\sum_{p=1}^{P} B_{p,s}x_{p,group1}=$$

$$\tilde{\Delta}_{s}= \sum_{p=1}^{P} B_{p,s}\left( x_{p,group2}-x_{p,group1} \right)$$

Because the standard logistic distribution has a standard deviation of ~1.81, we divide the latent mean difference by the standard deviation to calculate a standardised mean difference.

Because our explanatory variables are all dummy-coded, this procedure can be demonstrated with a simple example. We found that RCADS repression scores are elevated by 0.34 standard deviations in the medium food insecurity condition relative to the low food insecurity condition. If we work out the differences in the reference grid values for medium – low groups $\left( x_{p,group2}-x_{p,group1} \right)$ we see that differences are 0 except for the dummy variables for “sometimes” on the food insecurity questions, which are equal to 1. The latent mean difference then reduces to the sum of the beta coefficients for the three sometimes dummy variables (Food Bank – Sometimes, Unable to afford to eat – Sometimes, Go to bed hungry – Sometimes). We then divide the latent mean difference by the logistic standard deviation (~1.81). In practice, we repeat this procedure for each MCMC draw of the regression coefficients and then calculate posterior means and highest density continuous credible intervals. If we use the means of the regression coefficients to demonstrate, we see that we get a similar SMD:

$\mathrm{SMD}= \frac{\beta_{FoodBankSometimes}+\beta_{AffordToEatSometimes}+\beta_{HungrySometimes}}{1.81}=\frac{-.06+.26+.42}{1.81}= .34$

**Cell Probabilities (**$\boldsymbol{\pi}_{\boldsymbol{1}}\boldsymbol{,}\boldsymbol{\pi}_{\boldsymbol{2}}\boldsymbol{, ...,}\boldsymbol{\pi}_{\boldsymbol{K}}\boldsymbol{)}$


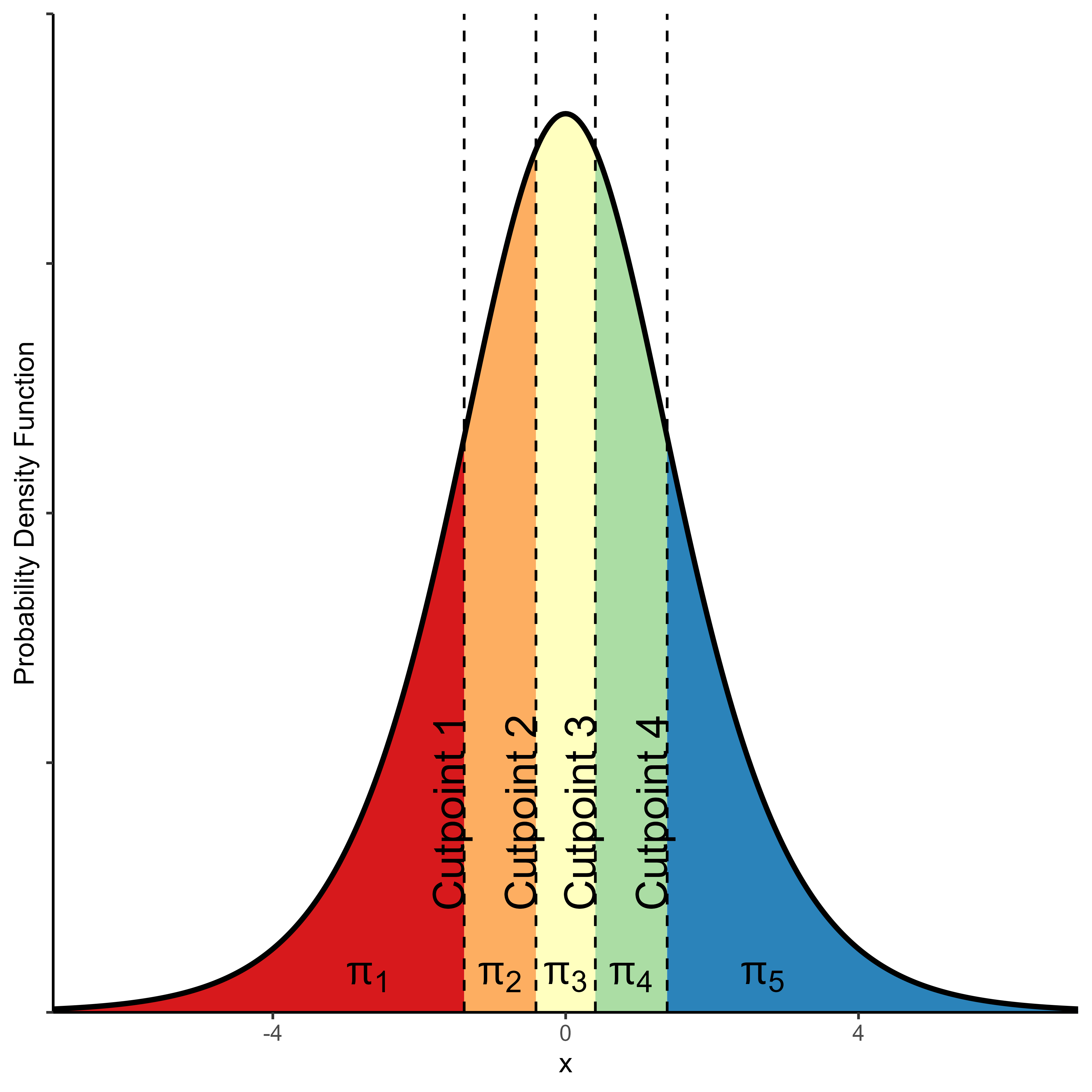


Initially, we planned to use the R package brms implementation of the cumulative-probit regression model. However, further testing showed that this function would fail to initialise when there are many levels to the outcome variable. Further investigation suggested that logit models tended to fit faster and with fewer problems than probit models, but a helpful suggestion by Professor Frank Harrell^[[1]](#footnote-2)^ was to reparameterize the model so that priors are specified on the “Cell Probabilities” rather than directly on the cutpoints.

The cell probabilities, denoted by the pi symbol ($\pi_{1}, \pi_{2}, ...,\pi_{K})$, refer to the areas under the logistic probability density function between each cutpoint when the linear predictor term is set to 0. These can be interpreted as the predicted probability of each response category when the linear predictor term is set to 0. We set Dirichlet prior distributions on the vector of cell probabilities ($\pi_{1}, \pi_{2}, ...,\pi_{K}\sim Dir\left( \boldsymbol{\alpha} \right)$) similar to the rmsb R package. The cutpoints are then calculated by adding the relevant cell probabilities and applying the quantile function for the standard logistic distribution function. The cutpoints are then used in the likelihood function.

$$\mathrm{cutpoint}_{j}=\mathrm{logit}\left( \sum_{j=1}^{j} \pi_{j} \right)$$

**Likelihood & Priors**

For an ordinal outcome $Y_{i}$, with *K* ordered levels, each level is represented by integers 1, 2, …, *K*. Let *N* be the total number of participants. For each participant *i,* their outcome $Y_{i}$ is modelled as:

$$Y_{i}\sim\mathrm{OrderedLogistic}\left( \boldsymbol{\eta}_{\boldsymbol{i}},\boldsymbol{c} \right)$$

The Ordered Logistic Distribution is described in the [stan documentation](https://mc-stan.org/docs/2_18/functions-reference/ordered-logistic-distribution.html). $\boldsymbol{c}$ is the vector of $K-1$ cutpoints. The value of the linear predictor for participant *i* is given by:

$$\eta_{i}=\mathbf{x}_{\mathbf{i}}\boldsymbol{\cdot}\boldsymbol{\beta}+\boldsymbol{\mu}\left[ j_{i} \right]$$

Where $\mathbf{x}_{\mathbf{i}}$ is a vector of data values on *P* explanatory variables for participant *i*. $\boldsymbol{\beta}$ is a vector of regression coefficients of length *P*. ***μ*** is a vector of *J* random intercepts for each school. Each school is assigned a unique integer identification code from 1 to *J*. The vector *j*, of length *N*, contains the school codes for all participants, with $j_{i}$ indicating participant *i* ’s school.$\boldsymbol{\mu}\left[ j_{i} \right]$ is the random school intercept for participant *i*.

Cell probabilities ($\pi)$ are also converted to cut points ($c)$ as follows:

$$c_{j}=\mathrm{logit}\left( \sum_{j=1}^{j} \pi_{j} \right)$$

Priors for the model parameters:

$$\boldsymbol{\mu}=z\times\text{sd}_{1}$$

$$\text{sd}_{1}\sim\text{Half-StudentT}\left( \nu=3,\sigma=2.5 \right),\quad\text{sd}_{1}>0$$

$$z\sim\text{Normal}\left( \mu=0,\sigma=1 \right)$$

$$\boldsymbol{\beta}_{\boldsymbol{1}},\boldsymbol{\beta}_{\boldsymbol{2}},\ldots,\boldsymbol{\beta}_{\boldsymbol{P}}\sim\text{StudentT}\left( \nu=3,\mu=0,\sigma=10 \right)$$

$$\text{conc}=\frac{1}{0.8+0.35\max\left( K,3 \right)}$$

$$\pi\sim\text{Dirichlet}\left( \underset{\text{ }\text{K}\text{ times}}{\underbrace{\text{conc},\text{conc},\ldots,\text{conc}}} \right)$$

## Supplement S8) Research question 2: ordinal regression full results table

Estimates (Est), highest-density 99% credible intervals (LB, UB) and probability of direction (PD) estimates for each ordinal logistic regression parameter across all models. Robust effects (PD > 99.5%) are highlighted in green.

|  | RCADS-11  Depression | | | | RCADS-11  Anxiety | | | | Adolescent Wellbeing SWEMWBS | | | | Positive Thoughts | | | | Loneliness | | | | Child Wellbeing SCWBS | | | |
| --- | --- | --- | --- | --- | --- | --- | --- | --- | --- | --- | --- | --- | --- | --- | --- | --- | --- | --- | --- | --- | --- | --- | --- | --- |
|  | Est | LB | UB | PD | Est | LB | UB | PD | Est | LB | UB | PD | Est | LB | UB | PD | Est | LB | UB | PD | Est | LB | UB | PD |
| Random Intercept (Logit Scale) | | | | | | | | | | | | | | | | | | | | | | | | |
| SD(School Random Intercept) | .18 | .14 | .22 | 1.00 | .18 | .14 | .23 | 1.00 | .15 | .11 | .20 | 1.00 | .14 | .10 | .19 | 1.00 | .20 | .15 | .27 | 1.00 | .29 | .20 | .38 | 1.00 |
| Regression Coefficients (Logit Scale) | | | | | | | | | | | | | | | | | | | | | | | | |
| Food Bank - Sometimes | -.06 | -.17 | .05 | .92 | .00 | -.11 | .12 | .52 | -.10 | -.25 | .04 | .96 | -.04 | -.20 | .11 | .76 | -.12 | -.26 | .02 | .98 | .04 | -.14 | .21 | .73 |
| Food Bank - Often | -.01 | -.25 | .22 | .57 | -.04 | -.28 | .19 | .69 | -.18 | -.48 | .13 | .93 | -.04 | -.36 | .29 | .61 | -.01 | -.30 | .28 | .53 | .78 | .40 | 1.16 | 1.00 |
| Unable to afford to eat - Sometimes | .26 | .14 | .38 | 1.00 | .09 | -.03 | .21 | .98 | -.23 | -.36 | -.10 | 1.00 | -.13 | -.27 | .01 | .99 | .24 | .12 | .38 | 1.00 | -.15 | -.49 | .20 | .87 |
| Unable to afford to eat - Often | .10 | -.10 | .29 | .90 | -.03 | -.22 | .16 | .64 | -.19 | -.41 | .03 | .99 | -.07 | -.30 | .16 | .78 | .14 | -.07 | .34 | .95 | .60 | .11 | 1.10 | 1.00 |
| Go to bed hungry - Sometimes | .42 | .29 | .57 | 1.00 | .32 | .18 | .46 | 1.00 | -.34 | -.52 | -.18 | 1.00 | -.29 | -.48 | -.11 | 1.00 | .27 | .10 | .44 | 1.00 | -.26 | -.50 | -.03 | 1.00 |
| Go to bed hungry - Often | .83 | .57 | 1.10 | 1.00 | .72 | .45 | 1.00 | 1.00 | -.42 | -.75 | -.08 | 1.00 | -.44 | -.82 | -.08 | 1.00 | .23 | -.09 | .55 | .97 | -.65 | -1.10 | -.20 | 1.00 |
| Gender - Male | -.69 | -.74 | -.63 | 1.00 | -1.03 | -1.08 | -.97 | 1.00 | .76 | .70 | .83 | 1.00 | .69 | .62 | .76 | 1.00 | -.77 | -.83 | -.71 | 1.00 | .39 | .28 | .49 | 1.00 |
| Gender –Gender Diverse | 1.05 | .82 | 1.29 | 1.00 | .78 | .56 | 1.01 | 1.00 | -.71 | -.95 | -.47 | 1.00 | -.93 | -1.18 | -.69 | 1.00 | .82 | .57 | 1.07 | 1.00 | -1.07 | -1.91 | -.23 | 1.00 |
| Ethnicity - Mixed | .03 | -.08 | .14 | .75 | -.07 | -.18 | .04 | .95 | .01 | -.11 | .14 | .58 | .04 | -.09 | .17 | .77 | .03 | -.09 | .16 | .75 | .03 | -.20 | .27 | .64 |
| Ethnicity - Asian | -.14 | -.23 | -.05 | 1.00 | -.23 | -.32 | -.14 | 1.00 | .13 | .03 | .24 | 1.00 | .11 | .00 | .21 | 1.00 | -.15 | -.26 | -.05 | 1.00 | .12 | -.07 | .31 | .95 |
| Ethnicity - Black | -.15 | -.29 | -.02 | 1.00 | -.25 | -.38 | -.11 | 1.00 | .26 | .11 | .41 | 1.00 | .38 | .22 | .55 | 1.00 | -.11 | -.26 | .03 | .98 | .00 | -.32 | .32 | .52 |
| Ethnicity - Other | -.12 | -.26 | .01 | .99 | -.23 | -.37 | -.10 | 1.00 | .06 | -.09 | .22 | .85 | .20 | .04 | .35 | 1.00 | -.19 | -.34 | -.04 | 1.00 | -.15 | -.41 | .11 | .92 |
| Born in UK | -.06 | -.14 | .03 | .96 | .02 | -.06 | .10 | .72 | -.03 | -.12 | .07 | .75 | .05 | -.05 | .16 | .90 | .10 | .00 | .19 | .99 | .12 | -.06 | .30 | .96 |
| Parent born in UK | .02 | -.07 | .10 | .71 | -.05 | -.14 | .03 | .95 | .08 | -.01 | .17 | .99 | .05 | -.06 | .15 | .87 | .03 | -.06 | .13 | .79 | .07 | -.10 | .23 | .84 |
| X1430 - Often | 1.23 | 1.12 | 1.34 | 1.00 | 1.43 | 1.32 | 1.53 | 1.00 | -.82 | -.94 | -.69 | 1.00 | -.70 | -.83 | -.56 | 1.00 | 1.27 | 1.14 | 1.39 | 1.00 | -1.08 | -1.28 | -.87 | 1.00 |
| X1430 - Sometimes | .66 | .60 | .72 | 1.00 | .74 | .68 | .80 | 1.00 | -.48 | -.55 | -.41 | 1.00 | -.46 | -.54 | -.39 | 1.00 | .75 | .68 | .82 | 1.00 | -.58 | -.70 | -.47 | 1.00 |
| X1450 - Often | .38 | .19 | .59 | 1.00 | .27 | .07 | .48 | 1.00 | -.06 | -.29 | .18 | .74 | -.17 | -.41 | .08 | .96 | .27 | .05 | .49 | 1.00 | -.11 | -.52 | .29 | .77 |
| X1450 - Sometimes | .34 | .25 | .43 | 1.00 | .25 | .16 | .35 | 1.00 | -.15 | -.26 | -.04 | 1.00 | -.19 | -.30 | -.07 | 1.00 | .27 | .16 | .38 | 1.00 | -.30 | -.48 | -.12 | 1.00 |
| X1460 - Often | -.06 | -.23 | .10 | .84 | .03 | -.14 | .20 | .67 | .14 | -.06 | .33 | .96 | .06 | -.14 | .26 | .78 | -.12 | -.32 | .07 | .94 | -.12 | -.44 | .21 | .82 |
| X1460 - Some | .13 | .02 | .24 | 1.00 | .17 | .06 | .28 | 1.00 | -.04 | -.16 | .09 | .78 | -.13 | -.26 | .00 | .99 | .06 | -.06 | .19 | .91 | -.37 | -.61 | -.14 | 1.00 |
| X1480 - Often | .66 | .53 | .80 | 1.00 | .53 | .40 | .67 | 1.00 | -.61 | -.76 | -.45 | 1.00 | -.40 | -.56 | -.23 | 1.00 | .63 | .48 | .79 | 1.00 | -.54 | -.79 | -.29 | 1.00 |
| X1480 - Sometimes | .55 | .46 | .63 | 1.00 | .51 | .42 | .59 | 1.00 | -.50 | -.60 | -.40 | 1.00 | -.45 | -.56 | -.34 | 1.00 | .52 | .42 | .62 | 1.00 | -.57 | -.72 | -.42 | 1.00 |
| X1490 - Often | .15 | -.06 | .37 | .97 | .13 | -.10 | .35 | .93 | -.19 | -.47 | .08 | .97 | -.10 | -.38 | .19 | .82 | .04 | -.21 | .30 | .66 | -.11 | -.49 | .28 | .76 |
| X1490 - Sometimes | .22 | .12 | .32 | 1.00 | .18 | .08 | .28 | 1.00 | -.20 | -.32 | -.08 | 1.00 | -.16 | -.29 | -.03 | 1.00 | .18 | .06 | .30 | 1.00 | -.32 | -.48 | -.15 | 1.00 |
| School Y6 | .12 | .02 | .22 | 1.00 | .02 | -.09 | .12 | .66 |  |  |  |  |  |  |  |  |  |  |  |  | -.29 | -.39 | -.18 | 1.00 |
| School Y7 | .33 | .21 | .45 | 1.00 | .09 | -.03 | .21 | .97 |  |  |  |  |  |  |  |  |  |  |  |  |  |  |  | 1.00 |
| School Y8 | .62 | .50 | .74 | 1.00 | .23 | .11 | .36 | 1.00 | -.18 | -.27 | -.10 | 1.00 | -.26 | -.36 | -.16 | 1.00 | .13 | .05 | .22 | 1.00 |  |  |  | 1.00 |
| School Y9 | .79 | .67 | .91 | 1.00 | .28 | .16 | .40 | 1.00 | -.31 | -.40 | -.22 | 1.00 | -.51 | -.60 | -.41 | 1.00 | .21 | .12 | .30 | 1.00 |  |  |  | 1.00 |
| School Y10 | .92 | .79 | 1.05 | 1.00 | .29 | .16 | .42 | 1.00 | -.42 | -.52 | -.32 | 1.00 | -.57 | -.67 | -.46 | 1.00 | .19 | .09 | .29 | 1.00 |  |  |  | 1.00 |
| School Y11 | 1.12 | .99 | 1.25 | 1.00 | .40 | .27 | .53 | 1.00 | -.57 | -.68 | -.47 | 1.00 | -.57 | -.68 | -.45 | 1.00 | .38 | .28 | .48 | 1.00 |  |  |  | 1.00 |
| School Y12 | 1.11 | .96 | 1.26 | 1.00 | .37 | .21 | .52 | 1.00 | -.49 | -.61 | -.36 | 1.00 | -.48 | -.62 | -.34 | 1.00 | .39 | .26 | .52 | 1.00 |  |  |  | 1.00 |
| School Y13 | 1.12 | .95 | 1.30 | 1.00 | .49 | .33 | .66 | 1.00 | -.58 | -.72 | -.43 | 1.00 | -.49 | -.64 | -.34 | 1.00 | .40 | .26 | .55 | 1.00 |  |  |  | 1.00 |
| π_1_ | .18 | .17 | .20 | 1.00 | .11 | .10 | .12 | 1.00 | .01 | .01 | .01 | 1.00 | .01 | .01 | .01 | 1.00 | .26 | .24 | .28 | 1.00 | .00 | .00 | .00 | 1.00 |
| π_2_ | .14 | .13 | .14 | 1.00 | .10 | .09 | .11 | 1.00 | .00 | .00 | .00 | 1.00 | .00 | .00 | .00 | 1.00 | .15 | .14 | .16 | 1.00 | .00 | .00 | .00 | 1.00 |
| π_3_ | .15 | .14 | .16 | 1.00 | .11 | .10 | .11 | 1.00 | .00 | .00 | .01 | 1.00 | .00 | .00 | .01 | 1.00 | .13 | .12 | .13 | 1.00 | .00 | .00 | .00 | 1.00 |
| π_4_ | .13 | .13 | .14 | 1.00 | .11 | .10 | .11 | 1.00 | .01 | .00 | .01 | 1.00 | .00 | .00 | .01 | 1.00 | .12 | .11 | .13 | 1.00 | .00 | .00 | .00 | 1.00 |
| π_5_ | .11 | .10 | .11 | 1.00 | .10 | .10 | .10 | 1.00 | .01 | .01 | .01 | 1.00 | .01 | .00 | .01 | 1.00 | .17 | .16 | .18 | 1.00 | .00 | .00 | .00 | 1.00 |
| π_6_ | .09 | .08 | .10 | 1.00 | .09 | .09 | .09 | 1.00 | .01 | .01 | .01 | 1.00 | .01 | .01 | .01 | 1.00 | .06 | .06 | .07 | 1.00 | .00 | .00 | .00 | 1.00 |
| π_7_ | .06 | .05 | .06 | 1.00 | .09 | .08 | .09 | 1.00 | .01 | .01 | .01 | 1.00 | .01 | .01 | .01 | 1.00 | .04 | .04 | .05 | 1.00 | .00 | .00 | .00 | 1.00 |
| π_8_ | .04 | .04 | .05 | 1.00 | .06 | .06 | .06 | 1.00 | .02 | .02 | .02 | 1.00 | .01 | .01 | .01 | 1.00 | .03 | .03 | .04 | 1.00 | .00 | .00 | .00 | 1.00 |
| π_9_ | .03 | .03 | .03 | 1.00 | .05 | .05 | .05 | 1.00 | .02 | .02 | .03 | 1.00 | .02 | .02 | .02 | 1.00 | .04 | .03 | .04 | 1.00 | .00 | .00 | .00 | 1.00 |
| π_10_ | .02 | .02 | .02 | 1.00 | .04 | .04 | .04 | 1.00 | .03 | .03 | .03 | 1.00 | .01 | .01 | .02 | 1.00 |  |  |  |  | .00 | .00 | .00 | 1.00 |
| π_11_ | .02 | .01 | .02 | 1.00 | .04 | .03 | .04 | 1.00 | .04 | .03 | .04 | 1.00 | .02 | .01 | .02 | 1.00 |  |  |  |  | .00 | .00 | .00 | 1.00 |
| π_12_ | .01 | .01 | .01 | 1.00 | .03 | .02 | .03 | 1.00 | .04 | .04 | .05 | 1.00 | .02 | .02 | .02 | 1.00 |  |  |  |  | .00 | .00 | .00 | 1.00 |
| π_13_ | .01 | .01 | .01 | 1.00 | .02 | .02 | .03 | 1.00 | .05 | .05 | .06 | 1.00 | .02 | .02 | .02 | 1.00 |  |  |  |  | .00 | .00 | .00 | 1.00 |
| π_14_ | .01 | .01 | .01 | 1.00 | .02 | .01 | .02 | 1.00 | .06 | .06 | .07 | 1.00 | .02 | .02 | .03 | 1.00 |  |  |  |  | .00 | .00 | .00 | 1.00 |
| π_15_ | .00 | .00 | .01 | 1.00 | .01 | .01 | .01 | 1.00 | .09 | .09 | .10 | 1.00 | .03 | .03 | .03 | 1.00 |  |  |  |  | .00 | .00 | .00 | 1.00 |
| π_16_ | .01 | .01 | .01 | 1.00 | .01 | .01 | .01 | 1.00 | .07 | .07 | .08 | 1.00 | .03 | .03 | .04 | 1.00 |  |  |  |  | .00 | .00 | .00 | 1.00 |
| π_17_ |  |  |  |  | .01 | .01 | .01 | 1.00 | .07 | .07 | .08 | 1.00 | .07 | .06 | .07 | 1.00 |  |  |  |  | .00 | .00 | .01 | 1.00 |
| π_18_ |  |  |  |  | .01 | .00 | .01 | 1.00 | .07 | .07 | .08 | 1.00 | .04 | .03 | .04 | 1.00 |  |  |  |  | .00 | .00 | .00 | 1.00 |
| π_19_ |  |  |  |  | .01 | .01 | .01 | 1.00 | .07 | .06 | .07 | 1.00 | .04 | .04 | .04 | 1.00 |  |  |  |  | .00 | .00 | .00 | 1.00 |
| π_20_ |  |  |  |  |  |  |  |  | .07 | .06 | .07 | 1.00 | .04 | .04 | .04 | 1.00 |  |  |  |  | .01 | .00 | .01 | 1.00 |
| π_21_ |  |  |  |  |  |  |  |  | .06 | .05 | .06 | 1.00 | .05 | .04 | .05 | 1.00 |  |  |  |  | .01 | .00 | .01 | 1.00 |
| π_22_ |  |  |  |  |  |  |  |  | .06 | .06 | .07 | 1.00 | .05 | .04 | .05 | 1.00 |  |  |  |  | .01 | .01 | .01 | 1.00 |
| π_23_ |  |  |  |  |  |  |  |  | .03 | .03 | .03 | 1.00 | .05 | .04 | .05 | 1.00 |  |  |  |  | .01 | .01 | .01 | 1.00 |
| π_24_ |  |  |  |  |  |  |  |  | .02 | .02 | .02 | 1.00 | .05 | .05 | .06 | 1.00 |  |  |  |  | .01 | .01 | .01 | 1.00 |
| π_25_ |  |  |  |  |  |  |  |  | .02 | .02 | .02 | 1.00 | .08 | .07 | .08 | 1.00 |  |  |  |  | .01 | .01 | .01 | 1.00 |
| π_26_ |  |  |  |  |  |  |  |  | .01 | .01 | .01 | 1.00 | .04 | .04 | .05 | 1.00 |  |  |  |  | .01 | .01 | .01 | 1.00 |
| π_27_ |  |  |  |  |  |  |  |  | .01 | .01 | .01 | 1.00 | .04 | .03 | .04 | 1.00 |  |  |  |  | .01 | .01 | .01 | 1.00 |
| π_28_ |  |  |  |  |  |  |  |  | .01 | .01 | .01 | 1.00 | .03 | .03 | .04 | 1.00 |  |  |  |  | .01 | .01 | .02 | 1.00 |
| π_29_ |  |  |  |  |  |  |  |  | .02 | .02 | .02 | 1.00 | .03 | .03 | .04 | 1.00 |  |  |  |  | .02 | .01 | .02 | 1.00 |
| π_30_ |  |  |  |  |  |  |  |  |  |  |  |  | .03 | .02 | .03 | 1.00 |  |  |  |  | .02 | .01 | .02 | 1.00 |
| π_31_ |  |  |  |  |  |  |  |  |  |  |  |  | .03 | .02 | .03 | 1.00 |  |  |  |  | .02 | .02 | .03 | 1.00 |
| π_32_ |  |  |  |  |  |  |  |  |  |  |  |  | .03 | .02 | .03 | 1.00 |  |  |  |  | .02 | .02 | .03 | 1.00 |
| π_33_ |  |  |  |  |  |  |  |  |  |  |  |  | .09 | .08 | .09 | 1.00 |  |  |  |  | .02 | .02 | .03 | 1.00 |
| π_34_ |  |  |  |  |  |  |  |  |  |  |  |  |  |  |  |  |  |  |  |  | .02 | .02 | .03 | 1.00 |
| π_35_ |  |  |  |  |  |  |  |  |  |  |  |  |  |  |  |  |  |  |  |  | .03 | .02 | .04 | 1.00 |
| π_36_ |  |  |  |  |  |  |  |  |  |  |  |  |  |  |  |  |  |  |  |  | .03 | .03 | .04 | 1.00 |
| π_37_ |  |  |  |  |  |  |  |  |  |  |  |  |  |  |  |  |  |  |  |  | .03 | .02 | .03 | 1.00 |
| π_38_ |  |  |  |  |  |  |  |  |  |  |  |  |  |  |  |  |  |  |  |  | .03 | .03 | .04 | 1.00 |
| π_39_ |  |  |  |  |  |  |  |  |  |  |  |  |  |  |  |  |  |  |  |  | .04 | .03 | .04 | 1.00 |
| π_40_ |  |  |  |  |  |  |  |  |  |  |  |  |  |  |  |  |  |  |  |  | .04 | .04 | .05 | 1.00 |
| π_41_ |  |  |  |  |  |  |  |  |  |  |  |  |  |  |  |  |  |  |  |  | .04 | .04 | .05 | 1.00 |
| π_42_ |  |  |  |  |  |  |  |  |  |  |  |  |  |  |  |  |  |  |  |  | .04 | .04 | .05 | 1.00 |
| π_43_ |  |  |  |  |  |  |  |  |  |  |  |  |  |  |  |  |  |  |  |  | .04 | .03 | .05 | 1.00 |
| π_44_ |  |  |  |  |  |  |  |  |  |  |  |  |  |  |  |  |  |  |  |  | .04 | .04 | .05 | 1.00 |
| π_45_ |  |  |  |  |  |  |  |  |  |  |  |  |  |  |  |  |  |  |  |  | .04 | .03 | .04 | 1.00 |
| π_46_ |  |  |  |  |  |  |  |  |  |  |  |  |  |  |  |  |  |  |  |  | .04 | .04 | .05 | 1.00 |
| π_47_ |  |  |  |  |  |  |  |  |  |  |  |  |  |  |  |  |  |  |  |  | .03 | .03 | .04 | 1.00 |
| π_48_ |  |  |  |  |  |  |  |  |  |  |  |  |  |  |  |  |  |  |  |  | .03 | .03 | .04 | 1.00 |
| π_49_ |  |  |  |  |  |  |  |  |  |  |  |  |  |  |  |  |  |  |  |  | .03 | .03 | .04 | 1.00 |
| π_50_ |  |  |  |  |  |  |  |  |  |  |  |  |  |  |  |  |  |  |  |  | .03 | .02 | .03 | 1.00 |
| π_51_ |  |  |  |  |  |  |  |  |  |  |  |  |  |  |  |  |  |  |  |  | .03 | .02 | .04 | 1.00 |
| π_52_ |  |  |  |  |  |  |  |  |  |  |  |  |  |  |  |  |  |  |  |  | .02 | .02 | .03 | 1.00 |
| π_53_ |  |  |  |  |  |  |  |  |  |  |  |  |  |  |  |  |  |  |  |  | .02 | .02 | .03 | 1.00 |
| π_54_ |  |  |  |  |  |  |  |  |  |  |  |  |  |  |  |  |  |  |  |  | .02 | .02 | .03 | 1.00 |
| π_55_ |  |  |  |  |  |  |  |  |  |  |  |  |  |  |  |  |  |  |  |  | .02 | .01 | .02 | 1.00 |
| π_56_ |  |  |  |  |  |  |  |  |  |  |  |  |  |  |  |  |  |  |  |  | .02 | .01 | .02 | 1.00 |
| π_57_ |  |  |  |  |  |  |  |  |  |  |  |  |  |  |  |  |  |  |  |  | .02 | .01 | .02 | 1.00 |
| π_58_ |  |  |  |  |  |  |  |  |  |  |  |  |  |  |  |  |  |  |  |  | .01 | .01 | .02 | 1.00 |
| π_59_ |  |  |  |  |  |  |  |  |  |  |  |  |  |  |  |  |  |  |  |  | .01 | .01 | .01 | 1.00 |
| π_60_ |  |  |  |  |  |  |  |  |  |  |  |  |  |  |  |  |  |  |  |  | .01 | .00 | .01 | 1.00 |
| π_61_ |  |  |  |  |  |  |  |  |  |  |  |  |  |  |  |  |  |  |  |  | .01 | .01 | .01 | 1.00 |
| Note. Regression coefficients with highly certain effects (PD > 99.5%, equivalent to p < .01) are highlighted in green. | | | | | | | | | | | | | | | | | | | | | | | | |

## Supplement S9) Research question 2: exploratory subgroup analyses using multilevel models

Results from the multilevels model allowing the effects of the food insecurity questions to differ between gender, ethnicity and year groups are presented below. The advantage of multilevel models is that subgroup effects are regularized (e.g., [Asalti, Hussey, Elson & Arslan, 2023](https://osf.io/preprints/psyarxiv/fcm3n/)). Due to the very small percentage of participants who sometimes or never responded to the food insecurity questions, our estimates are likely to be imprecise when trying to estimate effects within small subgroups in the OxWell survey.

Multilevel model results measuring the standardised mean difference on each outcome variable within different subgroups.


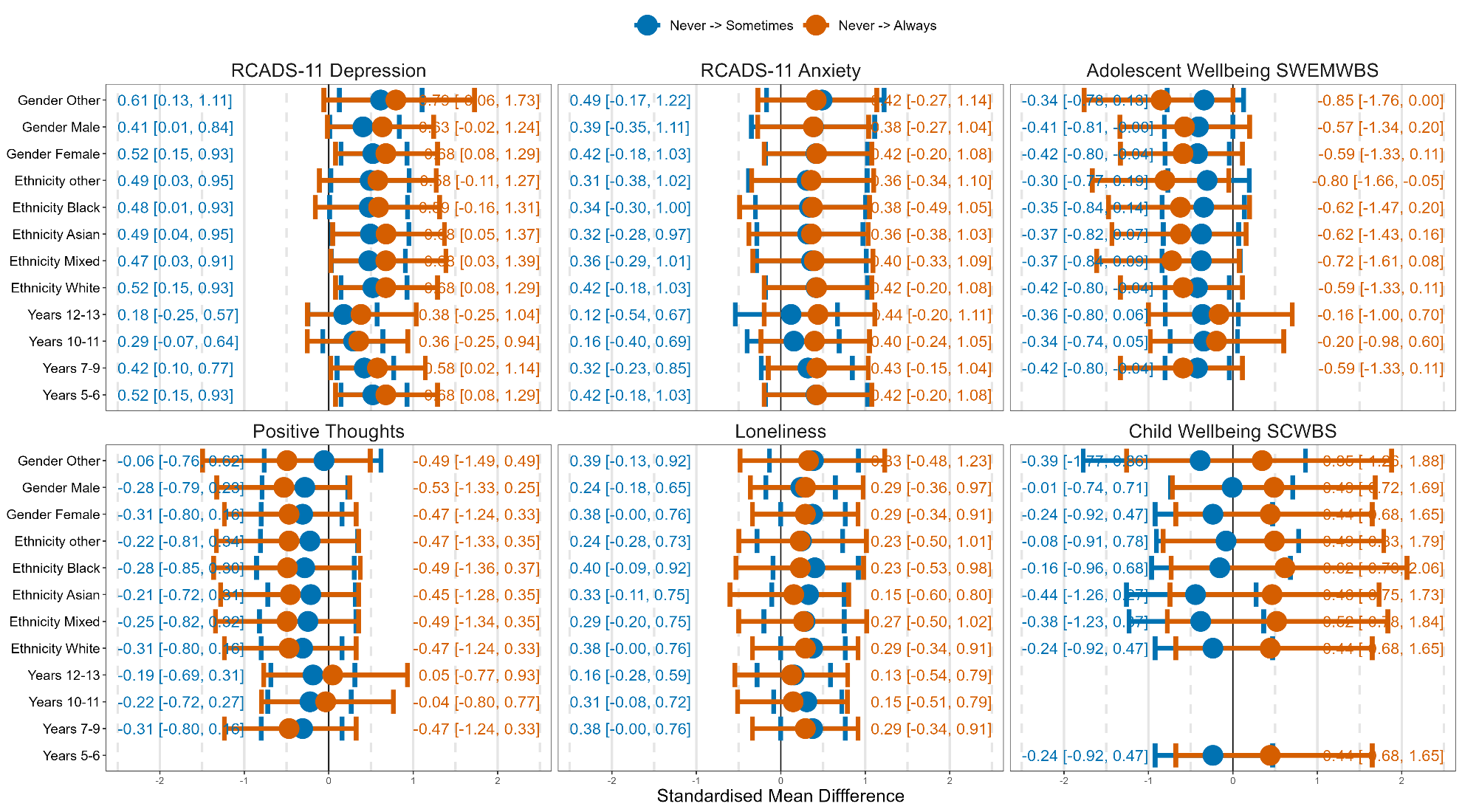


## Supplement S10) Research Question 2: distribution of each outcome variable stratified by food insecurity response

The figure below shows the distribution of each outcome variable for participants with different responses to each food insecurity question. Non-imputed data is used: missing data in the outcome variables are handled by calculating a mean score (of completed items) for each individual and multiplying the mean by the number of items. Each point represents the mean and errors (SE), and sample sizes (N) are shown for each condition.


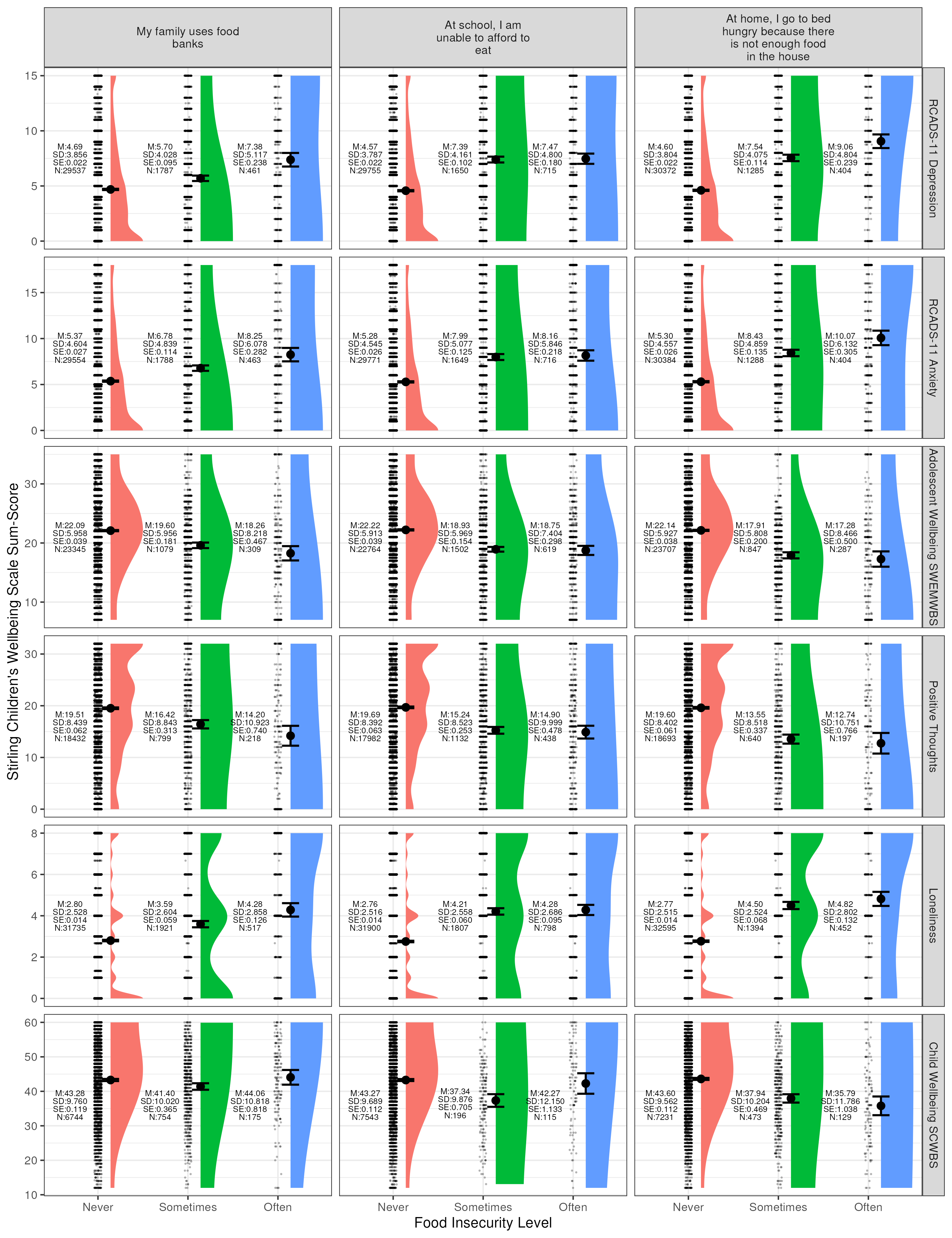


1. https://discourse.mc-stan.org/t/is-there-a-upper-limit-on-the-number-of-outcome-categories-for-cumulative-probit-ordinal-models/32935 [↑](#footnote-ref-2)
